# Integrating AI with Biosensors and Voltammetry for Neurotransmitter Detection and Quantification: A Systematic Review

**DOI:** 10.3390/bios15110729

**Published:** 2025-11-02

**Authors:** Ibrahim Moubarak Nchouwat Ndumgouo, Mohammad Zahir Uddin Chowdhury, Silvana Andreescu, Stephanie Schuckers

**Affiliations:** 1 Department of Electrical and Computer Engineering, Clarkson University, Potsdam, NY 13699, USA; mochowd@clarkson.edu; 2 Department of Chemistry and Biomolecular Science, Clarkson University, Potsdam, NY 13699, USA; eandrees@clarkson.edu; 3 Department of Computer Science, University of North Carolina-Charlotte, Charlotte, NC 28223, USA; sschucke@charlotte.edu

**Keywords:** artificial intelligence, deep learning, machine learning, pattern recognition, biosensors, voltammetry, neurotransmitters

## Abstract

Background: The accurate and timely diagnosis of neurodegenerative disorders such as Parkinson’s disease, Alzheimer’s disease, and major depressive disorder critically depends on real-time monitoring and precise interpretation of authentic neurotransmitter (NT) signal dynamics in complex biological fluids (CBFs), including cerebrospinal fluid. These NT dynamics are governed by both the type and concentration of NTs present in the CBFs. However, current biosensors face significant limitations in sensitivity and selectivity, thereby hindering reliable estimation (detection and quantification) of NTs. Though nanomaterials and bioenzymes have been utilized to modify sensor interfaces for enhanced performance, issues like signal convolution, electrode fouling, and inter-NT crosstalk persist. Objectives: This review aims to evaluate and synthesize current research on the use of artificial intelligence (AI), particularly machine learning (ML), pattern recognition (PR), and deep learning (DL), to improve the automated detection and quantification of neurotransmitters from complex biological fluids. Design: A systematic review of 33 peer-reviewed studies was conducted, focusing on the integration of AI methods in neurotransmitter estimation. The review includes an analysis of commonly studied NTs, the methodologies for their detection, data acquisition techniques, and the AI algorithms applied for signal processing and interpretation. Results: The studies reviewed demonstrate that AI-based approaches have shown considerable potential in overcoming traditional biosensor limitations by effectively deconvoluting complex, multiplexed NT signals. These techniques allow for more accurate NT estimation in real-time monitoring scenarios. The review categorizes AI methodologies by their application and performance in NT signal analysis. Conclusions: AI-enhanced NT monitoring represents a promising direction for advancing diagnostic and therapeutic capabilities in neurodegenerative diseases. Despite current challenges, such as sensor stability and NT interaction complexity, AI integration, particularly in applications like closed-loop deep brain stimulation (CLDBS), offers significant potential for more effective and personalized treatments.

## 1. Introduction

Neurotransmitters (NTs) serve as crucial biomarkers for neurodegenerative diseases such as Parkinson’s disease, Alzheimer’s disease, and major depressive disorder [1,2,3,4,5,6]. Understanding their neurological roles and accurately monitoring their levels in biological fluids like cerebrospinal fluids are essential for characterizing the onset and progression of these neurodegenerative diseases [1,2,3,4,5,6,7,8,9,10,11,12,13,14,15,16]. NT monitoring involves their detection, quantification, or estimation. Detection of NTs refers to their classification into specific categories, quantification involves predicting their concentration levels, and estimation refers to their simultaneous detection and quantification [1]. Traditional methods for monitoring NT dynamics include biosensors integrated with electrochemical techniques, such as differential pulse voltammetry (DPV) and fast-scan cyclic voltammetry (FSCV). These integrations have advanced our ability to study NT dynamics in both in vivo and in vitro settings, enhancing our understanding of NT dynamics in biological fluids. However, these integrations typically lack real-time capabilities and suffer from low spatial and temporal resolution, impairing their effectiveness in analyzing NT dynamics within complex biological environments [5,7]. Additionally, most biosensors are limited by poor selectivity and sensitivity, as they often struggle to differentiate and accurately quantify specific NTs [1,2,3]. Attempts have been made to improve biosensors’ selectivity and sensitivity by integrating nanomaterials and bioenzymes onto their functional surfaces, often through immobilization using biopolymers such as chitosan. Despite these advancements, challenges, such as signal convolution and interferences from complex NT interactions, electrode fouling, and inter-NT crosstalk, remain unresolved. To address these limitations, recent advancements have focused on integrating artificial intelligence (AI), particularly machine learning (ML), deep learning (DL), and pattern recognition (PR) with biosensors and electrochemical sensing techniques. These integrations have significantly enhanced real-time, in vivo detection and quantification of NTs, outperforming conventional methods used in isolation. AI models are effective at extracting meaningful features from complex and multidimensional data like voltammetric data, improving estimation accuracy and enabling adaptive, closed-loop systems for real-time NT monitoring [1]. Prospective applications of AI-enhanced NT monitoring include (1) closed-loop deep brain stimulation (CLDBS), which holds promise for advancing therapeutic interventions for neurodegenerative conditions including Parkinson’s by monitoring dopamine (DA) levels in cerebrospinal fluids; (2) diagnosis of conditions like pheochromocytoma and neuroblastoma by estimating urinary metabolites, such as vanillylmandelic acid (VMA) for norepinephrine (NE) and homovanillic acid (HVA) for dopamine (DA) from urine samples; and (3) detection of the onset or progression of carcinoid syndrome by monitoring serotonin (SE) levels in blood samples.

**Figure 1:**
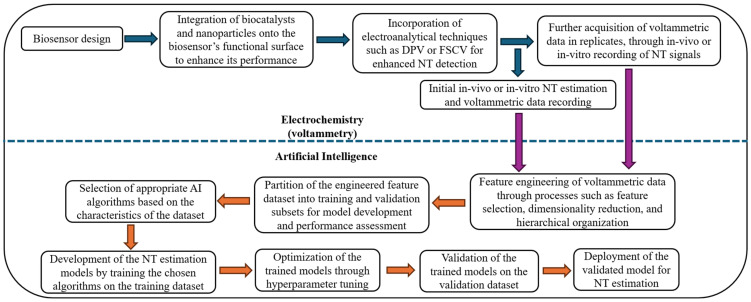
Block diagram summary of the integration of AI with biosensors and voltammetry for the estimation of neurotransmitters.

This review aims to systematically map existing research at the intersection of AI, biosensors, and voltammetry for NT detection and quantification and identify knowledge gaps by critically evaluating current studies in this field. It explores the methodologies employed, the types of biosensors and AI models utilized, key performance outcomes, and ongoing challenges in the field. Through this review, we aim to identify major trends, existing technological gaps, and future directions for the development of intelligent neurochemical sensing systems. The following introductory subsections provide an overview of key NTs, identify current knowledge gaps, outline estimation challenges, and examine how AI-driven approaches can address these issues. Figure 1 summarizes the integration of AI with biosensing and voltammetric methods for accurate NT estimation in biological fluids.

### 1.1. Synopsis of Neurotransmitters (NTs)

NTs are important biochemical messengers, synthesized and secreted in low and temporally varying physiological concentrations within the complex in vivo environments of the human body. These environments are mostly the intestines and various regions of the central nervous system (CNS), including the cerebrospinal fluids of the brain and spinal cord [1,2,3]. NTs coexist at equilibrium concentrations in the CNS, where they take part in synaptic transmission processes. During these processes, they act very fast before being instantaneously oxidized or recycled back into the nervous system after fulfilling their physiological, psychological, or behavioral roles [4,5,6,7]. Abrupt or undesired changes in NT concentrations can disrupt normal brain function and lead to severe or irreversible mental disorders such as Parkinson’s disease, Alzheimer’s disease, depression, and schizophrenia [1,2,3,4,5,7,8,9,10,12,14,15,16]. As NTs play vital roles in maintaining mental and physical health, they are, therefore, valuable biomarkers for these diseases, necessitating their real-time monitoring [1,2,3,4,5,7,9,10,12,14,15,16]. Dopamine (DA) is particularly well studied due to its significant role in neuromodulation and as a biomarker for diseases like Parkinson’s. It also influences various motivated behaviors [10,11,14,15,16]. Acetylcholine (ACH), serotonin (SE), epinephrine (EP), norepinephrine (NE), and glutamate (GT) are also under active investigation for their roles in mental and physical health [1,4,6,7,12,13,17,18,19]. However, a knowledge gap on the in vivo functions of these NTs persists, as they interact with each other, producing complex signals and crosstalk [1]. When NTs are detected and quantified using electrochemical methods, their signals are often multiplexed due to their complex mutual interactions, electrode “fouling”, and crosstalk, alongside interference from other neurochemicals and background noise present in the complex biological fluids [8]. This complexity makes accurate, real-time, in situ monitoring of NTs with conventional electrochemical biosensors very challenging [1,9]. To address these challenges, various neurochemical methods utilizing implantable biosensors (IBSs) have been proposed [10,11,12,13]. More than a hundred NT species have been identified, and research continues to discover more.

### 1.2. Knowledge Gap on Neurotransmitters and Their Estimation Using Biosensors

Our understanding of the intricate interactions between NTs and their impact on mental and physical health is still limited. This gap in knowledge is largely due to the absence of suitable sensors that can perform real-time, in vivo monitoring of NTs, particularly in the brain [18,19]. Currently, monitoring NT concentrations in real time, comprehending their complex functions in the brain, and assessing how their interactions affect health pose significant challenges for biomedical, bioinformatic, and chemometric research. To address these challenges, various biosensors have been developed, with more in the pipeline, to track the dynamic activities of NTs and measure the complex signal patterns they produce in vivo [4,20]. Biosensors are electrochemical transducers capable of converting physiological parameters like NT concentrations into measurable signals, typically electrical or optical in nature. They can be categorized based on their materials, such as metal nanoparticles, including carbon, polymers, and aptamers, or by the biocatalysts used for their fabrication, such as antibodies, DNA, microbes, or enzymes, which are applied to their functional surface for NT estimation [21,22,23,24,25]. Nanoparticles and biocatalysts are often immobilized on biosensors’ functional surfaces using biopolymers to improve the sensitivity and selectivity for NT estimation. Chitosan, a widely used biopolymer, is favored for its biocompatibility [26,27,28,29,30,31]. The application of biosensors and electrochemistry for in vivo NT estimation benefits from the distinct oxidation potentials of NTs, allowing for their sensing through neurochemical processes [21,22,23,25]. When estimating NTs in vivo, it is crucial to consider factors related to the biosensors, such as limit of detection (LOD), chemical selectivity, sensitivity, spatial resolution, temporal resolution, stability, reproducibility, and cost [22,23]. Traditional neurochemical methods for NT estimation include microdialysis, constant-potential amperometry, fast-scan cyclic voltammetry (FSCV), differential pulse voltammetry (DPV), and high-speed chronoamperometry. Among these, FSCV and DPV have demonstrated good chemical selectivity and sensitivity, with moderate temporal and spatial resolution when used with carbon fiber microelectrodes [32]. However, persistent challenges remain in the in vivo detection of NTs by electrochemical techniques.

### 1.3. Challenges in Neurotransmitter Estimation by Electrochemical Techniques and Their Mitigation

The challenges in employing electrochemical methods for the biorecognition of NTs arise from the fact that some electrochemically active NTs and other neurochemicals have similar chemical structures and oxidation potentials. These similarities lead to similar or comparable interactive chemical effects on biosensors, resulting in interferences among the recorded sensor signals. Such interference produces multiplexed signal patterns, crosstalk, and electrode “fouling” [1,2,3,33,34,35,36,37,38]. These issues compromise the sensitivity and selectivity of biosensors, making it difficult to accurately identify and quantify NTs within complex biological fluids [22,23]. The problem of low selectivity or insufficient qualitative detection for NTs by biosensors is addressed by incorporating NT-specific biocatalysts, such as antibodies, enzymes, or DNA, into the biosensors’ functional surfaces. These biocatalysts enhance the biosensors’ performances by catalyzing the oxidation of targeted NTs, which accelerates the detection process [21,22,23,25]. The catalytic oxidation of NTs lowers their detection potential and generates byproducts that can be detected electrochemically. For example, tyrosinase catalyzes the conversion of electrically inert DA into electroactive dopaquinone, which can be easily detected electrochemically [18,23]. Catecholamine NTs like glutamate, histamine, and acetylcholine are also electrochemically inert, complicating their selective detection with biosensors. To overcome this, NT-specific enzymes, such as glutamate oxidase for glutamate and acetylcholinesterase for acetylcholine, are used to catalyze their oxidation, enhancing their detection [10,18,19,20,21,22,23]. These enzymes lower the oxidation potential of NTs, improving detection speed and selectivity in complex biological fluids [10,23]. However, practical in vivo implementation, particularly in the brain, is challenging because some enzymes require substantial amounts of oxygen for their catalytic activity [10,18,22,23]. This oxygen is often scarce in the confined regions of brain tissue where biosensors are implanted. To address this issue, nanoparticles have been proposed to enhance the enzyme-catalyzed reactions in these confined areas [4,25,34,39]. These nanoparticles are functionalized on the surfaces of implantable biosensors along with NT-specific enzymes, all immobilized by a biocompatible matrix such as chitosan biopolymer. Chitosan is particularly suitable for in vivo applications due to its non-toxic, biodegradable, biocompatible nature and FDA approval [26,27,28,29,30,31]. The biocompatible matrix ensures that the nanoparticles and NT-specific enzymes remain in place on the biosensor’s functional surface within the intended region of implantation. The problem of low sensitivity or insufficient quantification of NTs by biosensors is addressed by reducing the size of the biosensors’ functional surfaces [12]. Miniaturizing these surfaces improves the spatial resolution of the biosensors through the enhancement of their effective functional surface area and detection limits. These moves improve the quantitative measurement of NTs. Additionally, incorporating nanoparticles onto the functional surfaces of the biosensors further boosts their spatial resolution, increasing the surface-area-to-volume ratio of the sensors. This amplifies the NT-signal transduction with higher sensitivity [23]. However, unresolved issues remain in the in vivo estimation of NTs using electrochemical techniques.

### 1.4. Unresolved Issues in NT Estimation by Electrochemical Techniques and Potential AI-Driven Solutions

Despite efforts to enhance the selectivity and sensitivity of biosensors, multiplexed signals from NT solutions still arise due to crosstalk between the NTs and other neurochemicals. To address this problem, various AI algorithms, statistical methods, and electrochemical techniques have been proposed to deconvolve these multiplexed signals to reveal “true” NT patterns [1,9,24,33,34,35,36,37,38,40,41,42,43,44,45,46,47,48,49,50,51,52,53,54,55,56,57,58,59,60,61,62]. Deconvolved NT signals have potential applications in future therapies, such as closed-loop deep brain stimulation therapies for neurological disorders like Parkinson’s disease [63,64,65,66,67,68,69,70]. AI algorithms have demonstrated superior accuracy in deconvolving NT patterns from complex in vitro and in vivo environments. AI is a broad domain within computer science that includes key areas such as machine learning (ML), deep learning (DL), pattern recognition (PR), and data science. Over the past decade, there has been a growing interest in applying AI to biomedical engineering, electrochemistry, bioinformatics, and chemometrics, although research in this area remains limited, as highlighted by reviews [71,72,73,74,75,76]. These reviews have primarily examined the integration of AI algorithms with biochemical and electrochemical sensors.

### 1.5. Related Reviews

Previous surveys have explored various methods for estimating NTs, including in vivo and in vitro approaches. However, none have specifically focused on the application of AI for the automatic detection and quantification of NTs. Table 1 provides a summary of previous surveys on neurotransmitter (NT) estimation, with emphasis on four key aspects: (1) NT analysis, indicating the structural and functional properties of NTs; (2) voltammetric sensing techniques, analyzing the electrochemical methods used in NT sensing; (3) simultaneous NT estimation, evaluating the concurrent detection and quantification of multiple NTs; and (4) application of artificial intelligence (AI)**,** assessing the integration of AI techniques into NT estimation.

### 1.6. Scope of This Review

This systematic review is unique in that it specifically addresses the use of ML, PR, and DL algorithms for the automatic detection and quantification of NTs in complex environments. We have explored the most studied NTs, the detection methods employed, the types of signals analyzed, and the ML and DL algorithms utilized. To conduct this review, we selected 33 non-duplicate papers based on established inclusion and exclusion criteria, focusing on the estimation of NTs through ML, PR, and DL techniques.

### 1.7. Organization of the Review

The rest of this review paper is structured as follows: Section 2 outlines the materials and methods used in this survey, including survey methodology, planning the survey, research questions (RQs), sources of review materials, and the search strategies. Section 3 details major themes used for conducting the survey, providing an overview of the most extensively studied NTs, discussing methods for estimating NTs in complex environments and techniques for deconvolving their multiplexed signal patterns, and exploring cutting-edge applications of AI algorithms in NT pattern recognition. Section 4 presents the survey outcomes, focusing on various techniques used for the estimation of NTs. Section 5 offers a general discussion of the review and addresses the challenges associated with applying AI algorithms to in vivo NT pattern recognition. It also discusses the potential applications of deconvolved multiplexed signal patterns, such as in therapies like deep brain stimulation for treating neurological disorders, including Parkinson’s disease. The paper ends with a conclusion and perspectives in Section 6.

## 2. Materials and Methods

The methodology and study design, summarized in Table 2, were systematically developed for this review based on a critical analysis of 33 peer-reviewed publications in the field, the key findings of which are presented in Table 3 and elaborated on in this section.

### 2.1. Review Methodology

This survey offers a comprehensive overview of cutting-edge ML, PR, and DL algorithms developed for the estimation of NTs from multiplexed sensor signals. It includes nearly all high-quality research on this topic. We follow the systematic review methodology outlined by Kitchenham et al. [80,81], which involves three key stages: planning, conducting, and reporting the survey.

### 2.2. Planning the Survey

**Figure 2:**
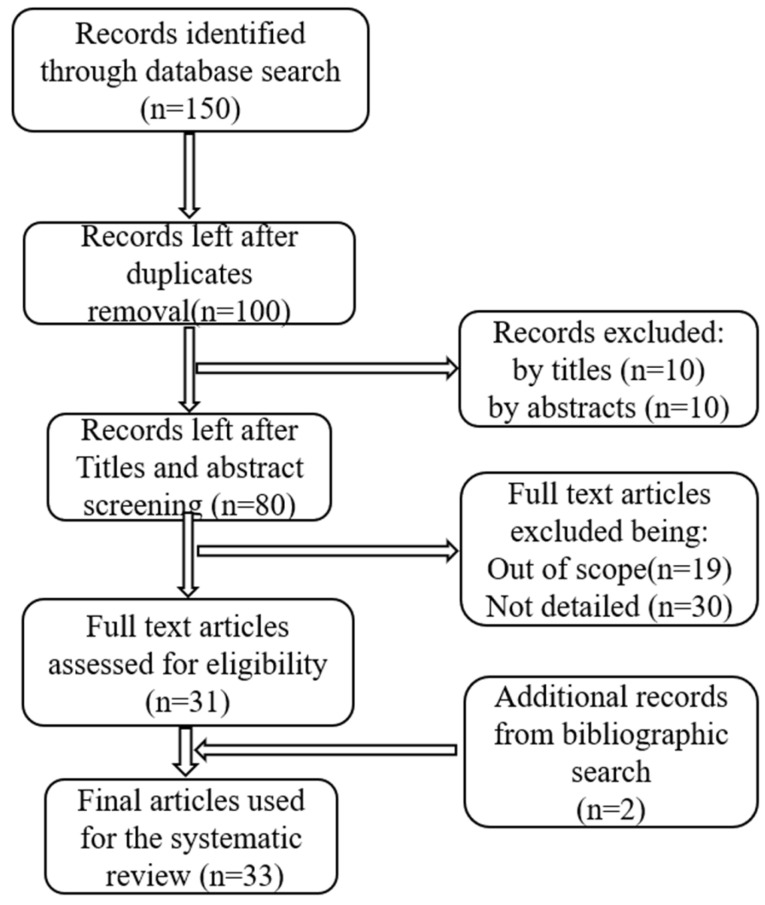
The PRISMA approach for selecting articles for the review.

We follow the Preferred Reporting Items for Systematic Reviews and Meta-Analyses (PRISMA) guidelines for this survey (Figure 2). PRISMA is commonly used to report systematic reviews because it provides a structured framework for thoroughly describing the review process. In line with PRISMA, we (a) define the research questions (RQs), (b) outline the sources of study materials, (c) establish the inclusion and exclusion criteria for the search strategies, and (d) present the results.

**Table 3:** Summary of the selected articles for the review.

Reference	Summary	Outcome	Publication Year
Sazonova et al. [1]	Used two pattern recognition techniques, principal component regression (PCR) and partial least squares regression (PLSR), with voltammetry to simultaneously estimate dopamine and serotonin, addressing signal overlap.	Achieved estimation accuracies ranging from 81% to 91% for DA and 91% to 100% for SE.	2009
Abbasi et al. [2]	Developed a quantum/carbon dot tricolor fluorescent probe to enable rapid, pattern-recognition-based discrimination of catecholamine NTs from ascorbic acid (AA) in urine with linear discriminant analysis (LDA).	Achieved 98% accuracy in NT discrimination using leave-many-out cross-validation.	2019
Xiaotong et al. [3]	Developed a metal nanoparticle-based nanozyme sensor array to enable pattern-recognition-driven discrimination of monoamine NTs in human serum using LDA and a hierarchical clustering algorithm (HCA).	Successfully discriminated monoamine NTs at varying concentrations with 100% accuracy.	2022
Jose et al. [9]	Used TinyML embedded in portable biosensors to discriminate NTs from uric acid (UA) and ascorbic acid (AA) interference for real-time applications.	Achieved NT discrimination accuracies of 98.1% using a 32-bit floating-point unit and 96.01% after 8-bit quantization.	2023
Martens et al. [21]	Predicted glutamate (GL) from whole-brain functional connectivity of the pregenual anterior cingulate cortex using elastic net (EN), PLSR, and HCA.	Achieved an R^2^ value (regression fit) of 0.143 and *p*-value (probability value) of less than 0.001 using EN for the prediction of GL.	2020
Nchouwat et al. [24]	Used nIRCat data to simultaneously detect and quantify age-dependent DA release in mouse brain slices using CatBoost regressor, which was later distillated to a kernelized ridge regressor (KRR) for improved performance.	Achieved a performance for the validation mean squared error (MSE) of 0.001 and an R^2^ value of 0.97 in estimating DA release.	2025
Salimian et al. [33]	Used UV–vis spectrophotometry coupled with net analyte system and PCR to simultaneously detect levodopa (LD) and carbidopa (CD) in mixtures, drugs, and breast milk.	Achieved mean recovery values of 96.86% for LD and 92.43% for CD using PCR, with corresponding mean squared prediction errors of 1.50 for LD and 7.14 for CD.	2022
Dowek et al. [34]	Developed a robust, pharmaceutical-grade method with PLSR to distinguish and quantify norepinephrine (NE) and epinephrine (EP).	Achieved R^2^ values of 0.95 and 0.91 for the quantification of EP and NE, respectively, with corresponding root mean square errors (RMSEs) of 5.47 for EP and 7.27 for NE.	2022
Jafarinejad et al. [35]	Designed an optical sensor array with three fluorescent dyes and pattern recognition to detect DA, EP, and NE by tracking changes in their emission when gold ions are present using LDA, artificial neural networks (ANNs), and multilinear regression (MLR).	Achieved an accuracy of 100% in discriminating NTs and their mixtures using LDA.	2020
Kallabis et al. [36]	Applied MLR, KRR, and Bayesian linear regression (BLR) models to quantify dopamine concentrations amid nonlinear variations induced by magnesium ion interactions.	Achieved a mean absolute percentage error of approximately 6–7% across all models, which is slightly above the experimental error observed in the absence of magnesium ions.	2024
Jafarinejad et al. [37]	Proposed a high-performance colorimetric sensor array and pattern recognition (PCA, LDA, and HCA) to detect and distinguish catecholamines (DA, EP, NE, and their mixtures) by their ability to reduce silver onto gold nanorods.	Achieved a discrimination accuracy of 100% for the individual NTs and their mixtures using LDA.	2017
Siamak et al. [38]	Utilized nIRCat imaging combined with machine learning models, support vector machine (SVM), and random forest (RF), to uncover distinct dopamine release patterns across different regions of mouse brains.	Achieved average detection accuracies of 55.5% and 83.2% using SVM and RF, respectively, in studies involving mice younger than 12 weeks.	2023
Komoto et al. [40]	Directly observed a single NT (DA, SE, NE, or their mixtures) by measuring tunneling current flowing through the single NT, using nanogap electrodes and XGBoost classifier.	Identified the spatial distribution patterns of NTs in the brain with high temporal resolution.	2020
Hoseok et al. [42]	Compared the performance of deep learning (DL) and principal component regression (PCR) in predicting NT concentrations, focusing on DA, SE, EP, and NE.	Demonstrated that DL slightly outperformed PCR for NT detection, achieving an average accuracy of 96.23% compared to 95.39% with PCR.	2022
Seongtak et al. [43]	Used deep learning to simultaneously estimate tonic DA and SE with high temporal resolution in vitro.	Achieved statistically significant accuracy (*p* < 0.001) for the in vitro estimation of DA and SE.	2023
Rantataro et al. [44]	Selectively detected DA and SE at nanomolar concentrations from complex in vitro systems in real time with electrochemical techniques.	Achieved an average R^2^ value of 0.99 for both DA and SE estimation using cyclic voltammetry (CV) and chronoamperometry.	2023
Buchanan et al. [45]	Used convolutional neural networks to evaluate SE neurochemistry in vivo.	Achieved statistically significant accuracy (*p* < 0.0001) for the in vivo estimation of SE.	2024
Simon et al. [46]	Focused on linear and quadratic regression models to describe an FPGA-based system for measuring NT concentrations on a multi-sensor platform, utilizing a visible-light optical spectrometer.	Achieved a mean training precision of 91.22% and a mean validation precision of 90.19% for NT estimation using quadratic regression.	2020
Doyun Kim et al. [47]	Automated cell detection method for TH-positive dopaminergic neurons in a mouse model of Parkinson’s disease using convolutional neural networks.	Successfully detected TH-positive dopaminergic neurons with a recall of 78.07%, precision of 74.46%, and an F1 score of 76.51%.	2023
Jian Lv et al. [48]	Developed a nanopipette method coupled with an XGBoost classifier to detect DA in single exosomes.	Achieved a classification accuracy of 99% for DA detection in single exosomes.	2023
Credico et al. [49]	Applied ML algorithms (LDA, XGBoost, and LightGBM) to identify phenotypic profile alterations of human dopaminergic neurons exposed to bisphenols and perfuoroalkyls.	Achieved classification accuracies ranging from 88% to 96.5% across the three algorithms.	2023
Arijit Pal Et al [50]	Detected DA using a machine-intelligent web app interface and a paper sensor modified with MoS2.	Achieved classification accuracy of 99%.	2023
Kammarchedu et al. [52]	Electrochemically detected NTs (DA, SE, EP, and NE) using a customizable machine learning-based multimodal system based on K-nearest neighbors (KNNR) and decision tree regressors (DTR).	Successfully differentiated between the four NTs and selectively detected each when independently present in complex media.	2023
Bang et al. [53]	showed that NE tracks emotional modulation of attention in human amygdala and estimated NE, SE, and DA in vivo using deep learning.	Achieved statistically significant accuracy (*p* < 0.001) for the in vivo estimation of NE, DA, and SE.	2023
Sanjeet et al. [54]	Simultaneously detected DA and SE in an optimized carbon thread-based miniaturized device using several ML algorithms.	Achieved an R^2^ value of 0.99 for both DA and SE estimation using a k-nearest neighbors regressor and a random forest regressor.	2024
Goyal et al. [55]	Applied voltammetry coupled with deep learning (DiscrimNet architecture) to estimate tonic concentrations of highly similar NTs (DA, SE, and NE) and their mixtures.	DiscrimNet accurately predicted changes in DA and SE levels, even in the presence of interfering substances like cocaine or oxycodone, demonstrating low RMSEs across all NTs.	2024
Unger et al. [56]	Analyzed the directed evolution of a selective and sensitive SE sensor using ML (random forest and generalized linear model).	Used ML to demonstrate the detection of SE release in freely moving mice during fear conditioning, social interactions, and sleep–wake transitions.	2020
Movassaghi1 et al. [57]	Simultaneously monitored SE and DA across timescales via rapid pulse voltammetry (RPV) coupled with partial least squares regression (PLSR).	Demonstrated that RPV-PLSR outperforms FSCV-PCR in the simultaneous monitoring of DA and SE.	2021
Zhang et al. [58]	Applied deep learning to automatically classify and predict NT (GABA, acetylcholine, and glutamate) synapses using electron microscopy.	Successfully identified NT synapses from EM images to construct a complete neuronal connectivity map, achieving 98% validation accuracy.	2022
Matsushita et al. [59]	Automatically identified phasic dopamine release using SVM.	Accurately identified phasic DA using automatically extracted patches, achieving 89.18% accuracy and a best F-measure of 77.23%.	2018
Matsushita et al. [60]	Improved the automatic identification of phasic dopamine release from fast-scan cyclic voltammetry data using convolutional neural networks (CNNs).	Achieved 97.66% accuracy in phasic DA detection using an end-to-end CNN object detection system based on YOLOv3.	2019
Xue et al. [61]	Introduced a deep learning–voltammetry platform for the selective analysis of three neurochemicals (ascorbate, DA, and sodium chloride) in live animal brains.	Selectively and simultaneously estimated neurochemicals with high spatial and temporal resolution.	2021
Nchouwat et al. [82]	Used PCR and PLSR for the simultaneous estimation of NTs, reducing complexity for SE and DA.	Simultaneously estimated DA and SE with 97.6% accuracy, while reducing the number of feature subsets required for the NT estimation.	2025

#### 2.2.1. Research Questions

In order to review the existing research in this field and identify knowledge gaps by critically evaluating current studies at the intersection of AI, biosensors, and voltammetry for NT detection and quantification, the following research questions were addressed for this systematic review:

RQ1: Which NTs were studied? This question explores the various types of NTs, including their electroactivity, chemical structures, functions, central nervous system locations, and receptor interactions.

RQ2: How were the multiplexed signal patterns of NTs recorded? This inquiry examines the different biosensors and neurochemical techniques used to capture multiplexed NT signals from complex biological fluids, as well as the experimental settings (in vitro or in vivo).

RQ3: What were the characteristics of the datasets? We will review the datasets based on their size, features, and authenticity.

RQ4: Which ML, PR, or DL algorithms were employed for NT estimation? This question will assess the AI algorithms used, including whether they were supervised or unsupervised, classification or regression, parametric or non-parametric, linear or nonlinear. It will also cover the ML workflows involved, such as feature selection, normalization/regularization, dimensionality reduction, model selection, model training–validation–testing, and the quality metrics used to evaluate the performance of the trained models.

#### 2.2.2. Sources of Study

A thorough search for high-quality research for this review was conducted from 2009 to May 2025 across several key repositories, including ACM Digital Library, Google Scholar, MDPI, ScienceDirect, Wiley Online Library, IEEE Xplore, Web of Science, Elsevier, Scopus, and Springer. Additionally, the search was expanded to include prominent conferences such as EMBS, where significant research in this field is actively presented and published.

#### 2.2.3. Search Strategy for the Review

The PRISMA approach was used to collect relevant materials for this review, as elaborated on in Figure 2. Only English-language papers were included, and the selection was guided by a set of free-text search terms: “estimation of neurotransmitters”, “pattern recognition of neurotransmitters”, “detection and prediction of neurotransmitters”, “automatic quantification of neurotransmitters”, and “neurotransmitters and chemometrics”. Boolean operators such as “and,” “or,” “-“, and “~” were employed to refine these search terms. Papers were selected based on the eligibility criteria outlined in Table 2, which details the standards for inclusion and exclusion according to the PRISMA methodology used in this review.

A search conducted in the specified repositories identified 150 relevant publications for this systematic review. After screening the titles, abstracts, full-text eligibility, scopes, and duplicates, 81 papers were selected. Of these, 49 were excluded for not meeting the established selection criteria. Ultimately, 31 research papers on the application of ML and DL algorithms for pattern recognition of NTs were included in the review. Additionally, 2 relevant studies found in the bibliographies of these papers were incorporated, bringing the total number of reviewed publications to 33, as presented in Figure 2.

**Figure 3:**
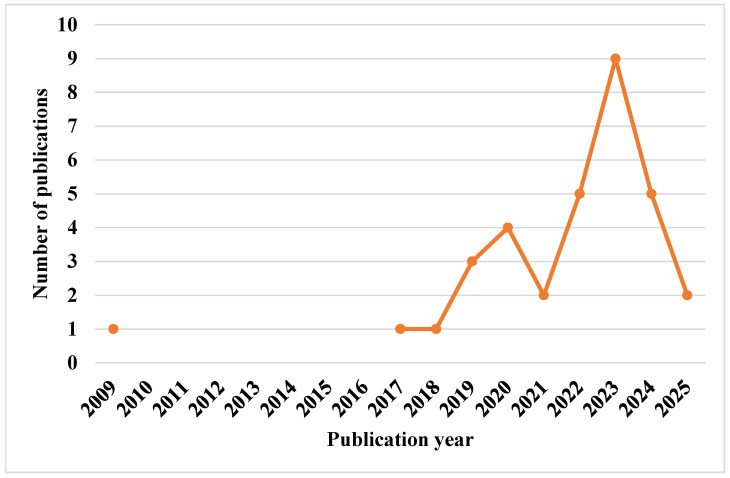
Publication trend using AI for estimating NTs from 2009 to May 2025.

Figure 2 illustrates the process for selecting the 33 final articles included in this systematic review, with Table 3 summarizing the purposes of these articles and years of publication. Figure 3 depicts the publication trends in this field from 2009 to May 2025.

Figure 3 illustrates that the lowest numbers of publications were in 2009, 2017, and 2018, with only one article published each year, while the highest number was recorded in 2023, with a peak of nine publications.

## 3. Conducting the Survey

This section provides a detailed explanation of the most studied NTs, their estimation using electrochemical techniques and AI-based approaches, the challenges associated with their accurate estimation, and the strategies implemented to address these challenges.

### 3.1. Neurotransmitters (NTs)

The most extensively studied NTs, due to their crucial roles in neurology and medicine, include dopamine (DA), serotonin (SE), epinephrine (EP), norepinephrine (NEP), GABA, glutamate (GL), and acetylcholine (ACH). They function as intermediaries in chemical synaptic transmission, facilitating communication within the central nervous system (CNS), between the CNS and the peripheral nervous system, and between neurons and other cell types [1,2,3,4,12,18,19,21,22,23,25]. They are released in temporally varying concentrations from presynaptic neurons and travel to postsynaptic receptors on other neurons or cell types, where they mediate neuro-signal conversion, amplification, and transmission [18,19,21,22,23,25].

**Figure 4:**
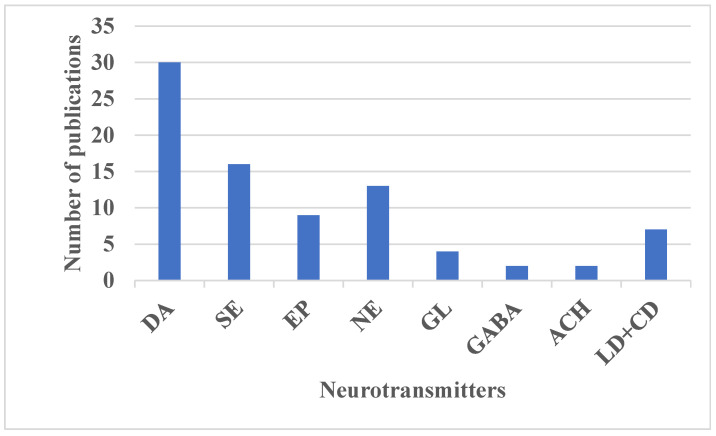
Frequency of NTs analyzed in papers related to AI.

The specific types and concentrations of NTs produced in the brain influence their regulatory functions, making them important biomarkers for various neurological disorders. For example, DA levels are associated with Parkinson’s disease [83,84,85,86,87,88,89,90,91,92,93,94] and depression [93,94], GL is linked to schizophrenia [24,51], and ACH is related to Alzheimer’s disease [62]. Changes in NT concentrations can affect a range of human cognitive functions, including emotions, thoughts, memories, learning, and movement [14,15,62]. To be classified as NTs, these chemicals must meet specific criteria: (1) they must be produced and released by the same neuron and stored at the presynaptic terminal; (2) they should induce specific responses in the postsynaptic neuron; (3) their external administration should replicate these effects; and (4) their action on the postsynaptic cell should be reversible through a specific mechanism [95]. The researched NTs, whose patterns have been recognized using various ML, PR, and DL algorithms, are discussed and summarized in Table 4, and the frequency of their analysis is depicted in Figure 4.

#### 3.1.1. Dopamine (DA)

DA, scientifically known as 4-(2-aminoethyl)-1,2-benzenediol, is a crucial monoamine NT extensively studied in neuroscience [83,84,85,86,87,88,89,90,91,92,93,94]. It acts as an excitatory neuromodulator, produced by dopaminergic neurons in key brain regions such as the substantia nigra, ventral tegmental area, and hypothalamus [85,86,87,88,89,90]. DA plays a significant role in various physiological functions within the central nervous system (CNS), directly or indirectly influencing pleasure, satisfaction, and motivation. It is essential for muscle coordination and movement control. The balance between DA and ACH is critical for maintaining clinical health [95]. Disruptions in DA levels can lead to several neurological disorders, including Parkinson’s disease, Huntington’s disease, drug addiction, and schizophrenia. Parkinson’s disease is often managed with treatments involving dopamine precursors, such as levodopa (LD) and carbidopa (CD), which mimic dopamine’s effects in the brain.

#### 3.1.2. Serotonin (SE)

SE, scientifically known as 5-hydroxytryptamine, is a key monoamine and excitatory neuromodulator. Approximately 95% of SE production occurs in the enterochromaffin cells of the gut, where the amino acid tryptophan, derived from food, is converted into SE with the assistance of the enzyme tryptophan hydroxylase [96,97,98]. In the brain, SE is synthesized by neurons in the rostral and caudal groups of the raphe nuclei. As an electrochemically active NT, SE inhibits the release of DA and GL and modulates the transmission of GL and GABA [99]. It plays a crucial role in regulating mood, sleep, and appetite. Imbalances in SE levels are linked to mood disorders such as depression and anxiety.

#### 3.1.3. Glutamate (GL)

GL, scientifically known as (2S)-2-aminopentanedioic acid, is an alpha-amino acid derived from glutamine and is predominantly found in the central nervous system (CNS). As the most abundant NT, it plays a crucial excitatory role in neural signaling. Its primary functions include amplifying neural signals within the CNS; facilitating long-term potentiation; supporting cognitive processes; and influencing motor, sensory, and autonomic activities [100]. GL operates through a complex mechanism. It is released into the synaptic cleft by presynaptic neurons and then activates two key receptors: alpha-amino-3-hydroxy-5-methyl-4-isoxazolepropionic acid and N-methyl-D-aspartate. These receptors mediate the influx of sodium and calcium ions into postsynaptic neurons. However, an imbalance or excess of GL can lead to excessive calcium influx, resulting in heightened neuronal firing and excitotoxicity. This imbalance may be linked to various neurological disorders, including multiple sclerosis, amyotrophic lateral sclerosis, and Parkinson’s disease [101]. Astrocytes play a critical role in maintaining glutamate homeostasis by regulating its levels in the CNS.

#### 3.1.4. Acetylcholine (ACH)

ACH, scientifically known as 2-acetoxy-N, N, N-trimethylethanaminium, is an NT derived from acetic acid and choline, and it is crucial for various bodily functions. In the peripheral nervous system, it facilitates muscle contraction and supports autonomic nervous system functions. Within the central nervous system, acetylcholine is vital for cognitive processes such as memory and learning, and it also influences attention, arousal, and the sleep–wake cycle. Disruptions in ACH levels due to dysfunctions in cholinergic pathways are linked to conditions like Alzheimer’s disease and hallucinations [62].

#### 3.1.5. Epinephrine (EP) and Norepinephrine (NEP)

EP (adrenaline), scientifically known as 4-[(1R)-1-hydroxy-2-(methylamino) ethylbenzene-1,2-diol, and NEP (noradrenaline), scientifically known as (R)-4-(2-Amino-1-hydroxyethyl) benzene-1,2-dio, are two key monoamines that function both as NTs and hormones. NEP serves as an electrochemically active neuromodulator and plays a crucial role in the autonomic nervous system’s “fight-or-flight” response, impacting both the sympathetic and parasympathetic systems [95]. Neurons producing NEP are primarily located in the locus coeruleus and project to various brain regions, including the limbic system. Functionally, NEP is involved in regulating arousal, alertness, sensory signal detection, emotions, memory, learning, and attention [102]. In contrast, EP neurons are found in different brain regions, such as the lateral tegmental system and medulla, and their role as neurotransmitters is less well understood. However, epinephrine is known to influence the “fight-or-flight” response by increasing the heart rate, promoting vasodilation, dilating pupils, and elevating blood sugar levels [95].

#### 3.1.6. Gamma-Aminobutyric Acid (GABA)

GABA, scientifically known as 4-aminobutanoic acid, is an amino acid NT produced at neural junctions through the conversion of GL into GABA, facilitated by the enzyme glutamate decarboxylase [99,103]. As an electrochemically active NT, GABA primarily functions as the brain’s main inhibitory NT. However, in its early developmental stages, GABA can be excitatory due to its role in inducing depolarization rather than hyperpolarization. This occurs because of the high chloride concentration in neurons during early development, which causes chloride to exit the cells rather than enter. In mature adults, the chloride concentration changes, leading to an inward flux of chloride and transforming GABA’s role from excitatory to inhibitory [95]. GABA’s primary role is to reduce neural excitability and maintain a balance between inhibitory and excitatory signals in the brain. Proper GABA levels are crucial for normal brain function and help prevent central nervous system issues such as behavioral disorders, sleep disturbances, epilepsy, and pain.

Table 4 provides an overview of the categories, locations, roles, and associated pathologies of the NTs examined in the studies selected for this systematic review. Figure 4 illustrates the frequency of research conducted on these NTs. Most of the publications investigated more than one NT. DA stands out as the most extensively researched NT, with 30 publications, followed by SE with 15, NE with 13, EP with 9, LD+CD with 7, GL with 4, GABA with 2, and ACH with 2 publications. DA’s prominence in research is attributed to its critical roles in the central nervous system and its status as a key biomarker for neurodegenerative disorders such as Parkinson’s disease, Huntington’s disease, depression, and schizophrenia [83,84,85,86,87,88,89,90,91,92,93,94]. However, the isolation of pure DA signals in vivo is challenging due to the multiplexing of its electrochemical signatures with those of other NTs and biomolecules within complex biological fluids.

### 3.2. Origin of Multiplexed Neurotransmitter Signals and Motivation of the Need for AI

**Figure 5:**
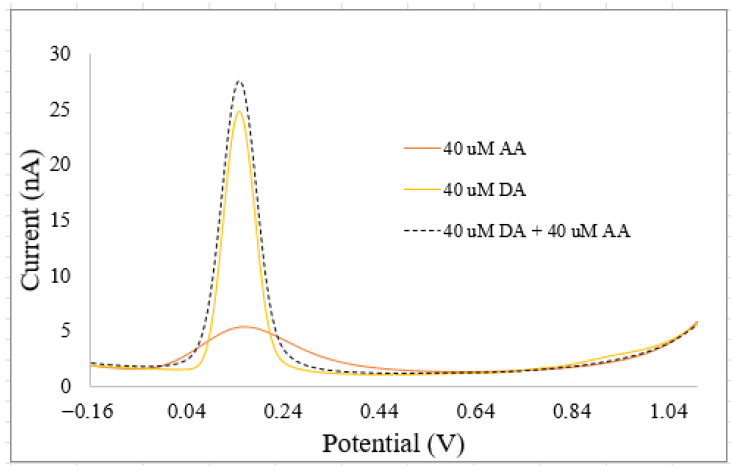
Voltammograms recorded for 40 μM DA, 40 μM ascorbic acid (AA), and a mixture containing 40 μM DA and 40 μM AA using custom-built electrodes with bare surfaces (without the incorporation of nanoparticles or bioenzymes). The oxidation peak currents for all three solutions appear at approximately the same potential (~0.14 V), which can be attributed to the structural similarity between AA and DA, resulting in overlapping electrochemical behavior. However, the peak current of the mixed solution is significantly amplified compared to either individual species, indicating a nonlinear response that does not correspond to the peak current response of pure DA or AA.

NTs coexist at equilibrium concentrations with other neurochemicals in biological fluids. Some of these NTs have similar chemical structures, while others have comparable sizes to other neurochemicals that are not NTs or exhibit similar chemical properties to metal ions [1,2,3]. These similarities lead to complex interactions between NTs, neurochemicals, and metal ions, causing biosensors to record multiplexed signals that do not correspond to the pure signals of any single NT, as demonstrated on Figure 5 for DA and ascorbic acid (AA), measured with a custom-built electrode. Ascorbic acid is a neurochemical that is assimilated as an NT due to its structural similarities, roles in the central nervous system, and its potential to interfere with sensor response patterns from NTs [2,3]. Sazonova et al. [1] explored how similar chemical structures of NTs affect biosensor performance, focusing on NT detection and concentration prediction using voltammetry and pattern recognition. Their study revealed that the patterns of DA and SE interfere with each other. They proposed AI models to differentiate these patterns in in vitro mixtures. Jose et al. [9] examined the impact of NT sizes like other neurochemicals, in addition to the effects of similar chemical structures. Their research aimed to enhance electrochemical biosensors with AI to improve DA detection in the presence of interfering substances like uric acid (UA) and ascorbic acid (AA). Kallabis et al. [36] investigated the influence of NTs with chemical properties like metal ions, focusing on quantifying DA amid magnesium ions, with the aid of machine learning tools. Salimian et al. [33] analyzed DA precursors using net analyte signal and principal component regression to quickly determine levodopa and carbidopa in pharmaceutical formulations and breast milk samples through a spectrophotometric method. Dowek et al. [34] studied the interaction between EP and NEP, using surface-enhanced Raman spectroscopy with gold nanoparticle suspensions for discriminative and quantitative analysis. EP and NEP also function as hormones in the body. Several biosensors with modified functional surfaces have been proposed to deconvolve multiplexed neurotransmitter (NT) signals, aiming to extract the true signals of interest from complex biological fluid. Table 5 summarizes the advantages of integrating AI with traditional biosensors for the estimation of NTs.

### 3.3. Electrochemical Biosensors

Biosensors are electrochemical transducers capable of converting physiological parameters like NT concentrations into measurable signals, typically electrical or optical in nature. Challenges such as low selectivity, sensitivity, detection speed, crosstalk, and sensor “fouling” frequently hinder their performance during biorecognition of NTs [1,2,3,33,34,35,36,37,38]. To address the issues of low selectivity and sensitivity of biosensors, specific biocatalysts and nanoparticles are employed, respectively, as discussed below and summarized in Table 6.

#### 3.3.1. Enhancement of Biosensors’ Selectivity

Specific biocatalysts are used to enhance biosensors’ selectivity and minimize fouling by enabling targeted oxidation of NTs. This process lowers the oxidation potentials of the NTs and converts them into detectable forms. For example, in a mixture of various NTs, the enzyme glutamate–oxidase (GmOx) selectively targets and oxidizes the electrochemically inert GL. This process is summarized by Equations (1) and (2).

Glutamate + O_2_ + (GmOx) ⟶ α-Ketoglutarate + NH_3_ + H_2_O_2_ (1)

H_2_O_2_ → O_2_ + 2H^+^ + 2e^−^ (2)

Glutamate biosensors use glutamate oxidase (GmOx) to catalyze the oxidation of electrically inert glutamate NT and the reduction of oxygen into hydrogen peroxide (H_2_O_2_). GL is not easily detected, but H_2_O_2_ is easily detected at a lower potential as it is converted into 2H^+^.

Biocatalysts commonly used are as follows:

1. Enzymes: Enzymes serve as biorecognition elements. They are NT-specific and catalyze a reaction with the target analyte. The resulting product is directly detected by the sensors at lower potentials, which enhances the selectivity for neurotransmitter detection.
2. Antibodies or Antigens: These also serve as bio-recognition elements on immunobiosensors. They bind specifically to the target analyte, and the resulting complex byproduct is detected.
3. DNA: DNA strands are used to detect complementary DNA sequences or specific genetic material when used on DNA biosensors.
4. Microbes: Microbes are functionalized on microbial biosensors, where the whole cells or parts of cells are used to detect analytes. These biosensors can be employed for environmental monitoring.
5. Light: Optical biosensors use light-based techniques for detection, such as fluorescence, luminescence, or surface plasmon resonance, for specific NT detection. Light of different frequencies is used for selective NT detection.
6. Pressure: Piezoelectric biosensors measure changes in mass on the sensor surface, typically using a quartz crystal microbalance during NT detection. Here, pressure is used as the discriminative parameter for the selective detection of the NTs in a complex mixture.

Njagi et al. [10] investigated amperometric detection of DA in vivo using an enzyme-based carbon fiber microbiosensor. They developed a novel implantable biosensor with carbon fiber material approximately 100 micromolar in diameter. The biosensor’s functional surface was engineered to be coated with tyrosinase biocatalysts and ceria-based metal oxides to detect DA in vivo. Chitosan was employed as the biopolymer to immobilize both the tyrosinase and the ceria-based metal oxides on the biosensor’s functional surface.

#### 3.3.2. Enhancement of Biosensors’ Sensitivity

Biosensors’ sensitivities are enhanced using nanoparticles, which facilitate the oxidation of NTs and enhance the activity of biocatalysts, as summarized in Table 6. These processes accelerate reactions at the biosensor’s functional surface. Biocatalysts and nanoparticles are immobilized on the electrode’s functional surfaces using biopolymers such as chitosan, known for its biocompatibility [26,27,28,29,30,31]. Most nanoparticles used are metals or metal oxides, valued for their unique chemical, catalytic, electrical, and optical properties, making them ideal for functionalizing biosensor surfaces [23]. Notable nanoparticles with small effective sizes include cerium (IV) oxide, gold, Fe_3_O_4_, ZnO, silver, SnO_2_, CuO/Mn_2_O_3_/silver nanoparticles, and platinum-doped cerium (IV) oxide [4,25,34,39,102,103]. The miniaturization of biosensors and nanoparticles enhances sensor sensitivity by extending their detection limits to nanomolar (nM) ranges [32]. This advancement improves the spatial resolution of implantable biosensors by increasing the surface-area-to-volume ratio, reducing the adsorption of NTs on sensor functional surfaces (a process known as passivation), and minimizing the diffusional delay of NTs at the sensors’ surfaces [23]. Additionally, it boosts the electron transfer capability at the surfaces of the biosensors. Enhancing biosensors’ selectivity and sensitivity has greatly improved their performance, but this advancement presents several challenges.

#### 3.3.3. Challenges Faced by Enhancing Biosensors’ Performance

Enhancing biosensors’ performance by using NT-specific enzymes, nanoparticles, and chitosan on sensor surfaces presents several challenges. These include: (1) the tendency for sensors to become unstable and degrade over time [23]; (2) the need for efficient enzyme functionalization on the biosensor surface to ensure high selectivity; (3) the requirement for large immobilization matrices, which are unsuitable for delicate applications like in the brain; and (4) the risk of enzyme conformational changes, which can reduce shelf-life, stability, and catalytic activity [22,23]. Addressing these issues is a key concern for researchers. To overcome these challenges, some are exploring noninvasive techniques for NT detection, such as the use of light for in vivo NT monitoring [46]. Nevertheless, improving the performance of biosensors enhances their transduction properties, enabling the generation of distinct and measurable electrical signals that can be effectively processed to estimate NT in vivo.

### 3.4. Transduced Signal Output from Biosensors and Electrochemical Measurement Techniques

In the biorecognition of NTs, biosensors convert NT concentration into measurable signals. These signals, which reflect the NT patterns, include variations in current, potential, and resistance (or combinations of these), as well as light, color, and pressure. The signals are derived from electrochemical techniques and correspond to amperometry, voltammetry, impedimetry, potentiometry, photometry, colorimetry, and piezometry, respectively. Electrochemical techniques are preferred because most NTs are either electrochemically active or can be converted into electrochemically active forms by biocatalysts, making them easily detectable. Among the electrochemical methods, voltammetry is the most prevalent due to its speed and ability to provide qualitative, quantitative, and real-time measurements of NTs [113]. The primary voltammetric techniques include cyclic voltammetry (CV), fast-scan cyclic voltammetry (FSCV), differential pulse voltammetry (DPV), and square wave voltammetry (SWV).

These voltammetric techniques differ in how they apply potential and measure current, which directly affects the shape, dimensionality, and information content of their signals. CV, the potential, is swept linearly forward and backward, producing smooth, continuous current–voltage curves that are typically 2D signals (current vs. potential). Linear sweep voltammetry (LSV) is similar but uses only a single sweep, giving simpler one-directional profiles. DPV and SWV superimpose pulses onto the base sweep, yielding discrete, high-resolution peaks that enhance sensitivity and noise discrimination, which is ideal for distinguishing closely spaced redox events. FSCV extends CV to high temporal resolution by sweeping potential rapidly and repeatedly, generating time-resolved 3D datasets (current vs. potential vs. time). For AI-based analysis, these differences matter because they dictate the data structure (continuous vs. discrete, 2D vs. 3D) and feature richness available for machine learning. CV and FSCV enable dynamic pattern recognition, while DPV and SWV offer sharper, lower-dimensional signals better suited for classification or quantification tasks.

**Figure 6:**
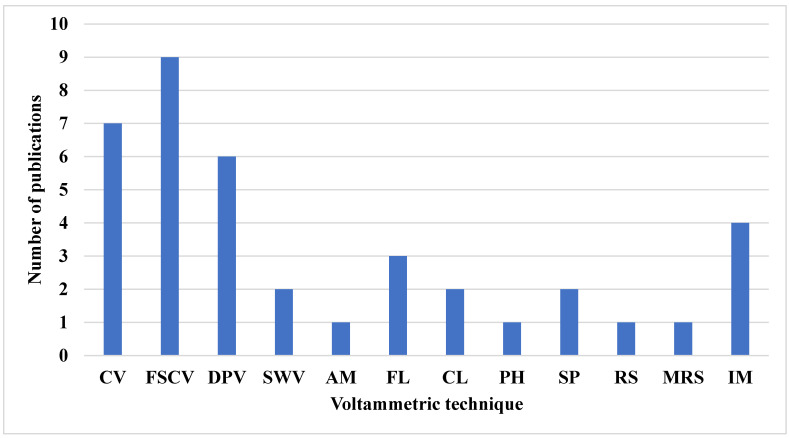
Frequency of application of voltammetric techniques in NT detection and quantification. Abbreviations are defined in Table 5.

Furthermore, the choice of these techniques depends on parameters such as response time, waveform, and detection limit. DPV is the slowest, with response times in seconds, whereas CV, FSCV, and SWV have response times in the sub-second range. The waveforms produced by DPV, FSCV, and SWV are staircase, triangular/sawtooth, and square, respectively. For in vitro detection of dopamine, SWV provides the lowest detection limit, with a value of 0.17 nanomolar [4,113]. Table 7 summarizes the electrochemical techniques used in the reviewed research papers, while Table 8 compares the use of voltammetric techniques and other electrochemical techniques. Figure 6 displays the frequency of their use over time. FSCV is noted as the most frequently used technique, appearing in nine publications, while amperometry (AM), photometry (PH), Raman spectroscopy (RS), and magnetic resonance spectroscopy (MRS) are nondestructive techniques used for NT estimation. Each appears in only one publication. This trend is largely attributed to FSCV’s ability to generate 2D or image data suitable for convolutional neural networks and deep learning analysis.

### 3.5. Artificial Intelligence (AI)

**Figure 7:**
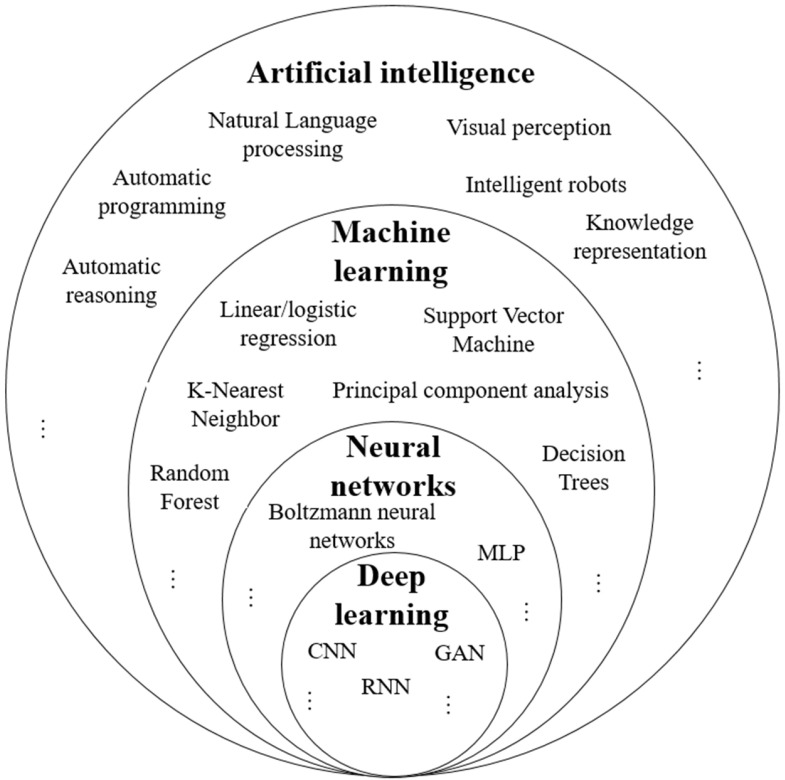
Proportions of the constitutive parts of AI with examples.

AI is a broad field of science that integrates various computer science disciplines, including cognitive computing, natural language processing, computer vision, machine learning, neural networks, deep learning, and data analytics [72,73,114,115,116,117]. Figure 7 presents the proportions of the constitutive parts of AI with some examples. AI leverages advanced hardware, software, and data to replicate and simulate human intelligence, judgment, and cognitive abilities. This allows AI systems to efficiently handle complex tasks such as decision-making, perception, and reasoning without human intervention [73]. AI algorithms can be classified into four categories, namely, supervised learning, semi-supervised learning, unsupervised learning, and reinforcement learning [113,114,115,118]. AI encompasses subfields like machine learning, pattern recognition, and deep learning.

#### 3.5.1. Supervised Learning

Supervised learning algorithms involve training AI models on a labeled dataset, where each input is paired with a correct output. The objective is to learn the relationship between inputs and outputs so the model can make accurate predictions on new, unseen data. Supervised learning tasks typically fall into two categories: classification, which involves predicting categorical labels (e.g., elephant, cows, or camels), and regression, which focuses on predicting continuous values (e.g., estimating house prices)

#### 3.5.2. Unsupervised Learning

Unsupervised learning algorithms identify patterns or structures in unlabeled datasets. Unlike supervised learning, where models learn from input–output pairs, unsupervised learning works with input data alone to uncover hidden relationships, groupings, or features. Common unsupervised techniques include clustering algorithms like K-means and hierarchical clustering, which group similar data points, and dimensionality reduction methods like principal component analysis (PCA), which simplify data by reducing its features while retaining key information. These algorithms are widely used in applications such as customer segmentation, anomaly detection, and data visualization. In 32 of the reviewed papers, the target or response of the model was clearly defined, as these studies employed supervised learning algorithms. None of the papers used semi-supervised learning algorithms, while three utilized unsupervised learning algorithms, as summarized in Table 9.

#### 3.5.3. Semi-Supervised Learning

Semi-supervised learning algorithms combine elements of both supervised and unsupervised learning. They utilize a small amount of labeled data alongside a larger portion of unlabeled data to enhance learning accuracy. This approach is particularly useful when large datasets are available but labeling them is costly or time-consuming. The algorithm first learns from the labeled data to identify patterns and then applies this knowledge to interpret the unlabeled data. Notable semi-supervised learning methods include self-training, co-training, and graph-based models. These techniques are widely used in real-world applications such as speech recognition, medical diagnosis, and image classification.

#### 3.5.4. Reinforcement Learning

**Figure 8:**
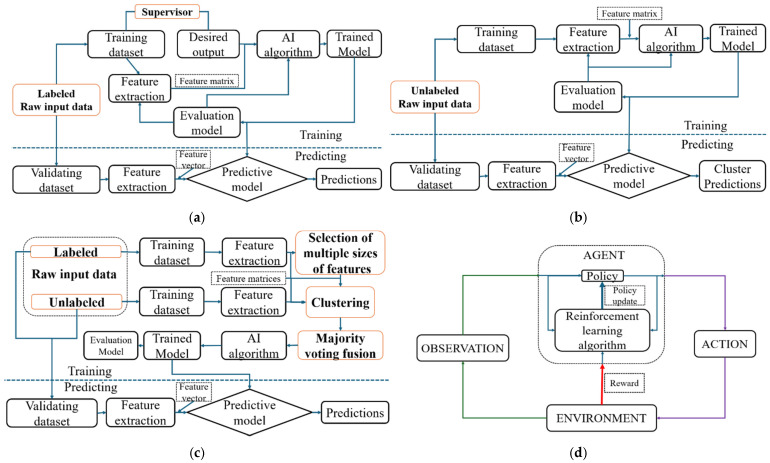
AI learning algorithms: (**a**) supervised learning algorithm, (**b**) unsupervised learning algorithm, (**c**) semi-supervised learning algorithm, and (**d**) reinforcement learning algorithm. Differences present in the learning algorithms are bolded in orange boxes. The arrows with different colors represent distinct information flow in the learning algorithms.

**Figure 9:**
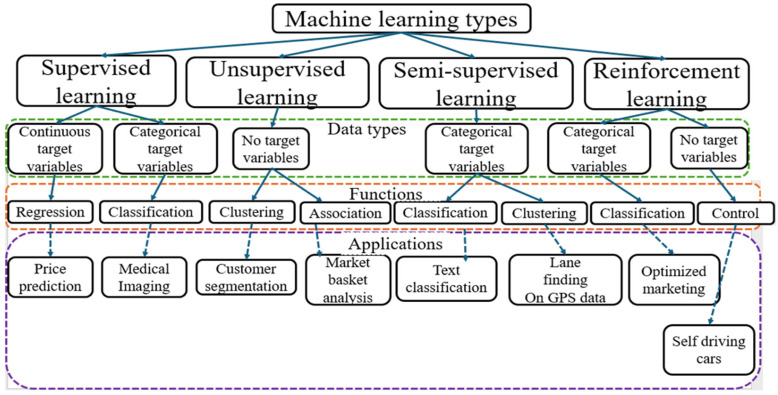
Machine learning types linked to different data types and functions. The colored boxes represent different parts of the learning algorithms that are categorized into data types, functions and applications.

Reinforcement learning algorithms are algorithms where an agent learns to make decisions by interacting with an environment. Instead of being explicitly taught, the agent learns through trial and error, receiving feedback in the form of rewards or penalties. The goal is to maximize cumulative reward over time by choosing the best actions based on its experiences. In these algorithms, the agent observes the state of the environment, takes an action, and receives a reward and a new state in response. Over time, it learns which actions lead to better outcomes. This approach is particularly useful in scenarios where it is difficult to define the correct answer for every situation in advance. Common applications of reinforcement learning include robotics, game playing, autonomous driving, and dynamic pricing systems. Table 9 summarizes the learning algorithms, Figure 8 illustrates them, and Figure 9 connects these algorithms to different data types, highlighting their functions and sample applications. For this review, 3 ([3,35,37]) publications focused on unsupervised learning, while the remaining 30 concentrated on supervised learning techniques in estimating NTs.

#### 3.5.5. Machine Learning Algorithms

ML is termed Artificial Narrow Intelligence (ANI). It is a branch of AI dedicated to creating algorithms and statistical models that enable computer systems to learn from data and make decisions without explicit programming [119,120]. The primary aim of ML is to identify general patterns within a training dataset to develop accurate decision rules for classification or regression tasks and then apply these rules to new, unseen data. ML has enhanced automation in decision-making across various domains, including image processing and data mining [119,120,121,122,123,124]. Figure 7 presents the proportion of ML in AI, and Figure 9 presents the types of ML, linking them to specific data types and illustrating their typical functions.

#### 3.5.6. Pattern Recognition (PR)

PR is a specialized area within ML focused on identifying and learning feature patterns in data. Unlike broader ML, which automates the process of feature learning, PR techniques are specifically designed for discerning feature patterns. In practical applications, PR can effectively distinguish and identify specific NTs or groups of NTs and has proven accurate in detecting and predicting concentrations of NTs such as SE and DA [1].

AI algorithms are applied to NT estimation in biological fluids or chemical mixtures based on the objective of the study, as follows:

1. Detection: Identifying and categorizing different NTs.
2. Quantification: Predicting the concentrations of known NTs.
3. Simultaneous detection and quantification: Determining both the types and quantities of NTs. The word “estimation” is also used interchangeably with simultaneous detection and quantification throughout this paper.

#### 3.5.7. Deep Learning

DL, particularly through convolutional neural networks (CNNs), addresses the limitations of traditional ML in feature learning by combining feature extraction blocks and artificial neural networks (ANNs) to perform tasks typically requiring human intelligence. The feature extraction blocks include input, convolution, activation (e.g., ReLU, sigmoid, and SoftMax), and pooling layers that introduce nonlinearity and enable the automatic learning of low- to high-level data features without human intervention. These blocks also support dimensionality reduction and feature selection, improving model training efficiency and reducing overfitting. The ANN forming the second part of the CNN performs classification or regression using deep neural networks composed of multiple interconnected layers inspired by multilayer perceptron (MLP) algorithms [45,47,53], and it is modeled after biological neurons in the brain. These networks are trained via backpropagation and optimized using gradient descent, though they require large, labeled datasets; significant computational resources; and careful hyperparameter tuning to avoid overfitting. To the best of our knowledge, the only research using DL and CNNs for pattern recognition in NT includes studies [43,45] and [45,47,53,76], respectively.

The use of AI to replicate and model human intelligence must adhere to stringent guidelines to ensure accurate analysis in critical areas such as medicine, biomedical science, and chemometrics [125]. For this systematic review, the chosen publications were required to follow these guidelines. These guidelines encompass the following:

a. Clarity of Aim

The papers reviewed had clearly defined problems and objectives, which facilitated the comparison of different algorithms across various datasets. This approach allowed us to rank AI algorithms based on their error rates for each dataset. For this review, we specifically targeted papers that explored AI applications for the automatic detection and quantification of NTs. Out of the 33 papers reviewed, 11 ([2,3,9,38,48,49,51,59,60,61,126]) concentrated on NT detection, while the remaining 22 addressed NT quantification or both detection and quantification (Table 3).

b. The Data

**Figure 10:**
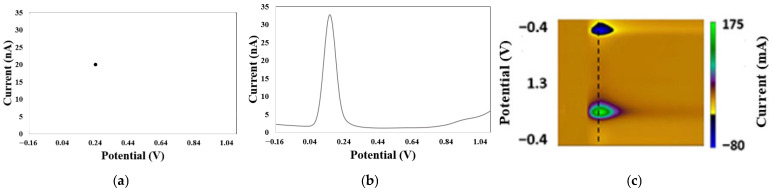
Example plots for the types of dimensionalities of electrochemical data with different units of measurement. (**a**) Zero-order data presented as a dot obtained by sampling the 1D voltammogram data of DA. This dot corresponds to discrete current and potential values of 20 nA and 0.24 V, respectively. (**b**) First-order data or 1D vectors. These are sample voltammograms of current vs. potentials for a triplicate of in vitro mixtures of serotonin and dopamine concentrations of 100 uM, recorded by DPV. (**c**) Second-order data. This is a pseudo-color plot of dopamine released in vivo after an FSCV experiment.

The electrochemical data considered in this review can be classified into two main types: time-dependent (time series) and potential-dependent. Time series data involve recording NT measurements over time, where time is the varying independent variable, while potential-dependent data involve recording NT measurements as the potential varies, with potential as the independent variable. In both cases, the current is typically the dependent variable. Time series data are commonly associated with in vivo studies, while voltage-dependent data are often linked to in vitro studies. Electrochemical data can also be categorized by their order: zero order, first order, or second order [76]. Zero-order data are point data or zero-dimensional (0D) data, usually recorded using potentiometric techniques. These are single-value data, and simple linear regression is commonly used for their analysis due to its straightforward computational procedure. First-order data are one-dimensional (1D) or one-dimensional vectors. These data represent the relationship between a dependent variable (such as current) and a varying independent variable (such as potential). Most electrochemical techniques, particularly in vivo, generate this type of data during the biorecognition of NTs. For instance, voltammetric techniques, which measure current as a function of applied potential, generate 1D data. Differential pulse voltammetry is a popular voltammetric technique for collecting 1D data. Various machine learning algorithms and artificial neural networks can be applied to analyze 1D data from the biorecognition of NTs. Second-order data are two-dimensional (2D) or image data, obtained by varying two independent variables simultaneously. Fast-scan cyclic voltammetry is a commonly used voltammetric technique for in vivo studies that produces pseudo-color plots of current as both potential and time vary during the biorecognition of NTs. Convolutional neural networks (CNNs) are particularly effective for analyzing 2D data, as they excel at extracting low-level features from these complex datasets. Due to the complexity of 1D and 2D data, which involve multiple dimensions, their analysis requires advanced computation. Dimensionality reduction techniques, such as linear discriminant analysis (LDA), principal component analysis (PCA), and partial least squares (PLS), are often employed to reduce dimensionality to 0D, simplifying the processing. Figure 10 illustrates different dimensionalities of electrochemical data.

In the publications reviewed, the types, sources, and recording processes of data used for detecting and quantifying NTs were crucial considerations. Setting clear objectives guided the data collection and analysis for optimal decision-making. Out of the studies examined, 9 ([21,38,45,47,48,49,51,59,60]) utilized data from real-world scenarios (in vivo), while the remaining 24 relied on synthetic data (in vitro). Real-world data is generally preferred over synthetic data for better generalization of AI models across various scenarios [119]. Most studies involved statistical analysis to ensure that their conclusions were objective and not due to chance. Key data preparation steps included curation, cleaning, and visualization. Visualization methods varied, with common approaches being histograms of error distributions, whisker-and-box plots, and range plots. Preprocessing steps typically involve handling missing values, encoding categorical variables, scaling numerical features, and splitting data into training and testing sets. Additionally, the studies clearly stated the hypotheses they aimed to test and verified whether the data supported these hypotheses.

c. Factors and Levels

Any parameter that influences the output of an AI model when altered is considered a factor. These factors include model hyperparameters, input features, and the training set, among others. The choice of these factors depends on the objectives of the experiments [76]. For example, factors can involve fixing an algorithm and optimizing its hyperparameters, comparing algorithms by “learning” one of them, or using multiple datasets.

Feature engineering is a crucial process in improving a model’s predictive performance. This process involves creating new features, selecting relevant ones, or transforming existing features [120,121]. Key aspects of feature engineering include feature selection, feature computation, dimensionality reduction, normalization, and hierarchy construction. Prior to selecting and designing an AI model, significant attention was given to choosing uncorrelated features that could effectively differentiate between classes of NTs. Techniques such as PCA, PLS [1,21,34,41,42], and LDA [2,3,35,37,49] were commonly used to reduce the dimensionalities of uncorrelated features. Table 10 and Table 11 summarize the various feature selection techniques and dimensionality reduction methods used in the reviewed papers, respectively, while Table 12 summarizes the feature categories that can be extracted from NT voltammograms.

d. The Procedure and Experimental Design

At this stage, the selection of the appropriate machine learning algorithm(s) is based on factors such as the type of problem (e.g., classification and regression), the characteristics of the data, and the performance criteria. Consideration may also be given to hyperparameter tuning and factorial designs. However, for the papers reviewed, factorial designs were not employed, as these studies had a clear understanding that the factors were independent and did not interact. This is because the number of experiment replications was determined by the dataset sizes [120]. Typically, replication sizes were kept small for large datasets, but this approach can sometimes make it challenging to compare distributions. For example, the assumption of a Gaussian distribution may not hold true for parametric tests. Generally, a portion of the data was reserved as a test set, while the rest was used for training and validation. Validation was performed multiple times through resampling the training data and averaging the model results from all samples. Table 13 summarizes the model selection and hyperparameter tuning techniques reviewed.

e. Performing the Experiment

The chosen model(s) are trained on the training dataset using the selected algorithm(s), which involves learning the patterns and relationships present in the data. Instead of training a new model, pre-trained models can also be utilized. Before conducting a large factorial experiment with numerous factors and levels, it is advisable to perform a few preliminary trials to ensure that all expectations are met. The algorithms were investigated thoroughly and impartially, with minimal bias from the experimenters when multiple algorithms were employed. Typically, it is best practice to have testers separate from developers for each model.

f. Performance Metrics

After training, the model’s performance is evaluated using a separate testing dataset or through cross-validation. Evaluation metrics vary depending on the specific problem but typically include accuracy, precision, recall, F1-score, and misclassification error for the detection of NTs and mean squared error, root mean squared error, mean absolute error, and R-squared values for quantification or simultaneous detection and quantification of NTs [72,76]. In cost-sensitive scenarios, both the output and system complexity are considered. Most of the studies reviewed primarily used accuracy as the quality metric for detection tasks, while mean squared error and mean absolute error were employed for quantification or simultaneous detection and quantification of NTs. The R-squared value was used to determine the best regression fits in quantification tasks. Table 14 and Table 15, below, summarize the differences between different metrics used for classification and regression problems, respectively.

**Table 14:** Summary of the metrics used for NT detection.

Metric	Formula	Focus	Range	Applied When	Sensitive To	References
Accuracy	$\frac{Correct\;predictions}{Total\;predictions}$ $\frac{TP+TN}{TP+TN+FP+FN}$	Overall correct predictions	[0, 1]	Classes are balanced and errors are equally weighted	Class imbalance	[1,2,3,9,33,35,38,48,49,50,58,59,60]
Precision	$\frac{TP}{TP+FP}$	Correctness of positive predictions	[0, 1]	False positives weigh more	False positives	[46]
Recall	$\frac{TP}{TP+FN}$	Finds actual positives	[0, 1]	False negatives weigh more	False negatives	[47]
F1-Score	$\frac{2.(Precision.Recall)}{Precision+Recall}$ $\frac{2TP}{2TP+FP+FN}$	Balance between precision and recall	[0, 1]	Classes are uneven with imbalanced datasets	Both FP and FN	[40,45,47,59,60]
Misclassification error	1-Accuracy $\frac{FP+FN}{TP+TN+FP+FN}$	Overall error	[0, 1]	An idea of the rate of error is needed	Class imbalance	N/A

where

- TP = true positives (correctly predicted positives). Here, the model said positive, and it is true.
- TN = true negatives (correctly predicted negatives). Here, the model said negative, and it is true.
- FP = false positives (incorrectly predicted positives). Here, the model said positive, but it is false.
- FN = false negatives (incorrectly predicted negatives). Here, the model said negative, but it is false.

**Table 15:** Summary of the metrics used for simultaneous NT detection and quantification.

Metric	Name	Formula	Focus	Range	Sensitiveness to Outliers	Interpretation	References
MSE	Mean Squared Error	$\frac{1}{n}\sum_{i=1}^{n}{\left(y_{i}-{ỹ}_{i}\right)}^{2}$	Penalizes large errors more heavily	[0, ∞]	Yes	Penalizes large errors with less intuitive units, as error is given as the square of the units of targets	[24,33,54]
RMSE	Root Mean Square Error	$\sqrt{\frac{1}{n}\sum_{i=1}^{n}{\left(y_{i}-{ỹ}_{i}\right)}^{2}}$	Penalizes large errors more heavily	[0, ∞]	Yes	Penalizes large errors more heavily with more intuitive units, as it gives error in the same units as the targets	[34,42,52,54,55]
MAE	Mean Absolute Error	$\frac{1}{n}\sum_{i=1}^{n}\left|y_{i}-{ỹ}_{i}\right|$	Robust and easy-to-understand average error	[0, ∞]	No	Less interpretable but error given with the same units as the targets	[36,54]
R^2^	Coefficient of Determination	$1-\frac{\sum {\left(y_{i}-{ỹ}_{i}\right)}^{2}}{\sum {\left(y_{i}-ỹ\right)}^{2}}$	Explains variance of the data	[−∞, 1]	Can be	Does not penalize large errors and explains how well models fit predictions	[1,21,24,33,54]

where

- $y_{i}$ = actual or true value;
- ${ỹ}_{i}$ = predicted value;
- *n* = number of data points.

g. Model Deployment

Once a satisfactory model has been developed and evaluated, it can be deployed into a production environment, where it will make predictions on new, unseen data. This deployment process may include creating APIs and integrating with other systems. After deployment, it is crucial to continuously monitor the model’s performance and retrain it as necessary to adapt to evolving data patterns. This ongoing maintenance ensures the model remains accurate and reliable in real-world conditions. José et al. [9] and Sanjeet et al. [54] were pioneers in proposing deployable models based on TinyML and Optimized Carbon Threaded-Based Miniaturized Devices, respectively.

#### 3.5.8. AI-Driven Solutions to Electrochemical Signal Multiplexing in Neurotransmitter Estimation

In voltammetry-based neurotransmitter detection in complex biological fluids, including the cerebrospinal fluids, peak overlap, background current drift, and redox potential shift of NT voltammograms resulting from NT dynamics present major analytical challenges that directly motivate AI integration with traditional electrochemical biosensors. Overlapping oxidation or reduction peaks from multiple neurotransmitters obscure individual signal features, making manual or traditional peak-based feature extraction unreliable [1,24,82]. Background current drift, caused by electrode fouling or electrolyte instability, distorts baselines over time and reduces the signal-to-noise ratio, complicating consistent training data generation [1,82]. Meanwhile, redox potential shifts due to electrode surface heterogeneity or environmental changes alter the apparent peak positions, undermining model robustness and generalization across sensors and recording sessions. Collectively, these nonlinear, time-dependent, and context-sensitive variations demand adaptive, data-driven approaches, such as machine learning, deep learning, and pattern recognition, to automatically learn invariant representations, compensate for drift, and enhance classification and quantification accuracy under realistic experimental variability [1,24,82]. Table 16 summarizes the link of each core challenge in using traditional electrochemical biosensors in isolation to its effect on feature extraction, impact on training robustness and model generalization, and motivation for AI integration.

## 4. Survey Outcome

This section presents details on the AI algorithms, methodological approaches, and key findings reported in the 33 peer-reviewed studies selected for this review, focusing on both in vivo and in vitro estimation of NTs.

### 4.1. Detection of Neurotransmitters

When both the types and concentrations of NTs in complex biological fluids are unknown, ML classification algorithms are used to identify the types of NTs present, without quantifying their concentrations [9]. Several algorithms have demonstrated effectiveness in distinguishing NT signals. These include supervised learning algorithms like LDA, support vector machines (SVMs), random forest (RF), extreme gradient boosting (XGBoost), and gradient boosting machine (GBM); unsupervised algorithms such as hierarchical clustering analysis (HCA); and deep learning (DL) algorithms, which have demonstrated superior accuracy compared to other approaches [2,3,9,38,48,49,51,59,60,61], as summarized on Table 16 and describe below.

#### 4.1.1. Application of Conventional Machine Learning Algorithms

a. Linear discriminant analysis

LDA is a supervised learning algorithm that discriminates between the different types of NTs in a mixture. It is a powerful tool used for dimensionality reduction in the data collected by the biosensors. LDA identifies linear combinations of original features in the data, thereby generating canonical factors [2,3]. These factors are linearly independent from each other. The linear independence of the factors minimizes within-class variance while maximizing between-class variance of the training data. This prevents the overfitting of the models when trained and tested with canonical factors.

Abbasi-Moayeda et al. [2], used LDA with two canonical factors (68.64% and 12.43%) for the rapid and visual detection of DA, NEP, and levodopa in a concentration range of 10–100 μM and discriminated these NTs from ascorbic acid in a concentration range of 40–80 μM with a cross-validation accuracy of 100%. To collect data, they designed a tricolor fluorescence probe incorporated into a single well with red quantum dots, blue carbon dots, and green quantum dots. The probe responded to three wavelengths of light, 450, 520, and 630 nm, and generated current as output. The data recorded was a matrix of 3 wavelengths (sensor elements) × 4 NTs × 9 concentrations × 3 replicates, providing a total of 324 data points.

Xiaotong et al. [3] used LDA with three different sets of canonical factors ((22.7%,62.4%), (88.9%, 10.8%), and (72.2%, 25.9%)) to detect DA, SE, EP, NEP, L-dopa, and DOPAC in concentrations ranging from 0.8 μM to 3 μM. Data was collected by using a colorimetric nanozyme sensor array with dendritic mesoporous silica embedded with metal nanoparticles (nanozymes). The nanozymes were silver, gold, and platinum nanoparticles. In total, 90 data points were recorded from a matrix of 3 nanozymes × 6 NTs × 5 replicates. The data collected were the changes in color fingerprints of the NTs that resulted from their inhibitory action on the catalytic effect of nanozymes.

b. Support vector machines

SVMs are supervised learning algorithms. They are effective in handling the complex nonlinear data recorded by biosensors during the biosensing of NTs. They provide robust generalizations of model performances. SVMs work by finding the optimal hyperplane that best separates data points belonging to different types of NTs in a high-dimensional space [38,55]. SVM’s aim is to maximize the margin, which is the distance between the hyperplane and the nearest data points corresponding to each NT. SVMs are robust in maintaining the tradeoff between bias and variance, reducing and thereby avoiding overfitting.

c. Random forest

RF is an ensemble learning method that builds strong predictive models by combining the predictions of multiple weak models, often decision trees [56]. RF is an algorithm used to solve problems with limited data recorded by biosensors during the biosensing of NTs. It is an example of bagging and ensemble algorithms. It is used to limit the risk of overfitting that may occur in decision trees, and it handles missing value data well. GBM and XGBoost, just like RF, are ensemble learning methods but have boosting properties. They differ from RF by training the data that were previously classified at each decision tree.

Siamak et al. [38] used SVM with a linear kernel and RF to detect DA release in vivo in mouse brains. The brains were stimulated with a single pulse of current with a strength of 0.1 or 0.3 mA. This generated time series data for DA at a concentration of approximately 2μM from two brain regions (where DA is released and where it is not). In total, 600 images were collected for every 200 frames of baseline fluorescence. Data was collected in triplicate with alternating pulse strengths. The Z-score was used to normalize the data, and leave-one-out cross-validation was used in training the models. An average detection accuracy of 55.5% was obtained with SVM, and 83.2% was obtained with RF when mice less than 12 weeks were used.

d. Hierarchical clustering algorithm

HCA is an unsupervised learning algorithm that groups data points of NTs together based on the similarities of their features or the distance between them [55]. The grouping is performed in a hierarchy of clusters in NT datasets in a tree-like structure called a dendrogram. References [2,3,35,37,55] used HCA to validate the in vitro results obtained from NT detection. They applied HCA for samples with fixtures of NTs with unknown concentrations or for in vivo environments like the brains of mice, where the concentrations of the NTs were unknown.

e. Embedded machine learning

The purpose of applying AI algorithms for automatic detection and quantification of NTs is to palliate the effects of interferences, crosstalk, and electrode “fouling” in the pattern recognition of NTs. DA is usually the target NT of importance. Jose et al. [9] used TinyML to enhance electrochemical biosensors by distinguishing DA from interfering substances (uric acid and ascorbic acid) using SWV data. They generated a dataset of 5492 samples through data augmentation and categorized it into four classes: Contaminated DA, Clean DA, PBS, and Unknown Chemical. Using a Keras-based deep learning model with four dense layers, they achieved high classification accuracies of 98.1% (32-bit) and 96.01% (8-bit) on an embedded system with minimal memory usage. The study demonstrated the feasibility of low-power AI for interference correction in DA detection. However, it was limited to four categories and did not reflect the complexity of real brain chemistry involving multiple neurotransmitters. Moreover, voltage-dependent data were processed as time series inputs, which may reduce accuracy in broader applications. The guidelines and procedures established by the work conducted by Jahangiri et al. [127] were not applied prior to assimilating the voltage-dependent data into time series data. Also, it is not clear why the window size chosen for their analyses was equal to the entire length of the patterns for all the categories. Furthermore, the work focused solely on detecting the categories without predicting their concentrations. However, the simultaneous detection and prediction of the concentration of the NTs is basic for the real-time in situ pattern recognition of dopamine. Lastly, the work does not explicitly or implicitly mention the pattern recognition algorithm applied in the proposed TinyML. This makes reproducibility unfeasible.

#### 4.1.2. Application of Deep Learning Algorithms

DA is released in the central nervous system through the activities of dopaminergic neurons, classified as tonic or phasic based on the speed and firing rates of these neurons. Tonic DA release occurs more slowly, over tens of seconds or minutes, while phasic DA release happens on a sub-second timescale, requiring fast and accurate monitoring systems for proper detection in vivo. Noninvasive detection techniques, such as imaging combined with deep learning, have shown promising results for monitoring DA.

Goyal et al. [55] proposed a deep learning model for detecting tonic DA from in vivo and in vitro voltammetry data. Matsushita et al. [59,60] developed CNNs for the automatic detection of phasic DA from FSCV data, achieving a performance of 98.31% with the CNN approach and 97.66% using an end-to-end object detection system with YOLOv3. The interaction between DA and SE also plays a role in determining susceptibility to Parkinson’s disease, as shown by Battaglia et al. [128]. Consequently, several studies have focused on the simultaneous detection and quantification of DA and SE [1,129], aiming to detect early symptoms of Parkinson’s disease with high accuracy for timely treatment.

This subsection analyzed how NTs are detected and classified into distinct categories. Table 15 summarizes the reviewed works that focused on the detection of NTs. A significant challenge in the detection of NTs lies in the simultaneous presence of multiple NTs at extremely low-equilibrium concentrations and other interfering species, like acids and metal ions, in biological fluids. Furthermore, the coexistence of multiple NTs poses considerable hindrances for obtaining real-time, species-specific quantitative information on the NTs. Consequently, these problems hinder the accurate classification of NTs into different categories, exacerbate the lack of real-time information on NT-specific quantities or concentrations in biological fluids, and impede a comprehensive understanding of the regulatory roles of NTs in the body. Therefore, precise and accurate quantification of NT concentrations is essential, as described next.

### 4.2. Quantification (Prediction of Concentrations) of Neurotransmitters

When the types of NTs in complex biological fluids are known, their individual concentrations can be predicted using regression algorithms. In this approach, the concentrations are estimated after identifying the specific NTs present. Supervised machine learning regression techniques proposed for this task include multivariate linear regression (MLR) [35], kernelized ridge regression (KRR) [36], Bayesian linear regression [36], simple linear regression [46], and quadratic regression [46]. Deep learning models have also been suggested for predicting NT concentrations. These algorithms are summarized in Table 13 and described below.

1. Linear Regression

Linear regression is a supervised learning algorithm commonly used to quantitatively determine the NTs present in a mixture from the voltammograms of currents recorded by biosensors. It is the simplest and fastest form of regression to implement and predicts continuous concentrations of NTs. In simple linear regression (Equation (3)), a linear function is established between an independent variable or feature (current) and a dependent variable or NT concentration [73,76]. In multivariate linear regression, a function is established between several independent features and dependent variables (Equation (4)).

y = wᵀx + b(3)

where y is the dependent variable or unknown concentration, x is the independent variable or scanning voltages, w is the weight matrix, and b is the bias term.

Y = XB + E(4)

Here, a multivariate linear regression function is established between several independent features (X) and dependent variables (Y), where matrix Y represents the dependent variables or the unknown NT concentrations; matrix X represents independent variables, corresponding to the scanning voltages (features); matrix B represents the weight coefficients; and matrix E represents the sum of the error terms and noise for each response, usually assumed to be normally distributed.

2. Quadratic regression

Quadratic regression models the relationship between the data variables of the NTs that are not linearly related but are curvilinear or have parabolic fits. A quadratic kernel is usually applied to recorded NT data to transform its original space to a quadratic space where a quadratic equation is fit to the data for more efficient analysis.

Sanjeet et al. [54] proposed a miniaturized hardware device using carbon threads for the simultaneous detection of DA and SE. Data was collected using cyclic voltammetry and differential pulse voltammetry in a potential range of −0.5 V to 1 V and a concentration range of 0.5 μM to 150 μM for DA and 0.5 μM to 200 μM for SE. Linear regression, random forest regression, GBM regression, and support vector regression were used to validate the results obtained from their proposed hardware device. The performance metrics used for the models were the mean absolute error (MAE) and the R-squared values. These metrics had values ranging from 16.25% to 41.56% for the MAE and 0.56 to 0.92 for the R-squared values.

3. Bayesian linear regression

Bayesian linear regression uses conditional probability to express the meaning of one feature from the recorded NT data as a linear combination of other features. In Bayesian linear regression, a Bayesian kernel is used on recorded NT data to transform its original space into a Bayesian space for Bayesian relationships to be established between the features.

4. Kernelized ridge regression

Kernelized ridge regression is a non-parametric technique used to estimate the conditional expectation of a random variable or features in the recorded NT data. In KRR, a kernel trick is used on the common ridge regression that has been modeled using features obtained by linear least squares techniques [73,76]. KRR is an extension of MLR obtained by adding a penalty on the sum of squares of the individual weights in MLR. KRR has an estimator that is like noisy Gaussian process regression [36].

Nchouwat et al. [24] developed a machine learning approach to automatically quantify dopamine (DA) release using near-infrared catecholamine nanosensor (nIRCat) data from mouse brain tissue across three developmental stages. By analyzing 251 image frames from the dorsolateral and dorsomedial striatum, a CatBoost regression model was trained, achieving high accuracy (R^2^ = 0.97, MSE = 0.001) within a ±10% prediction tolerance. For improved efficiency, the model was distilled into a kernelized ridge regression. This framework demonstrates strong potential for mapping age- and region-specific dopamine dynamics, supporting the advancement of targeted therapies for neurodegenerative diseases like Parkinson’s and depression.

Kallabis et al. [36] quantitatively determined DA in the presence of magnesium ions using kernel ridge regression (KRR) and Bayesian linear regression with an average error of 5.5%. Data was recorded using fluorine-doped tin oxide electrodes (FTOs) and differential pulse voltammetry. A total of 438 measurements of DA with concentrations ranging from 20 to 400 uM and magnesium with a concentration range of 0 to 600 uM were recorded with the FTOs in a potential range of −50 mV to + 350 mV vs. Ag/AgCl 1 M KCl. The features extracted from the 438 voltammograms were the peak currents, peak potentials, and the potential width at half peak currents. In total, 50% of the data was used for training and 50% for testing the models.

This subsection analyzed how accurate quantities or concentrations of NTs in complex biological fluids are predicted. Table 15 summarizes the reviewed works that focused on the quantification of NTs. A significant limitation of quantifying NT concentrations separately from their detection is that the process is time-consuming, costly, less accurate, and unsuitable for simultaneous analysis of multiple NTs in vivo. To address this challenge, the development of AI algorithms capable of analyzing multiple NTs in complex biological fluids is of utmost importance, as discussed next.

### 4.3. Simultaneous Detection and Quantification of Neurotransmitters

When the types and concentrations of multiple neurotransmitters (NTs) in complex biological fluids are unknown, it becomes necessary to simultaneously identify the individual NTs present and quantify their concentrations in order to effectively analyze their patterns. PCR, PLSR, and DL are commonly used pattern recognition algorithms for their simultaneous detection and quantification, as described below.

#### 4.3.1. Application of Principal Component Regression and Partial Least Squares Regression

PCR applies PCA to reduce feature dimensionality by extracting uncorrelated principal components, followed by regression fitting. However, PCR only maximizes variance in explanatory features, not the target variable, which limits its predictive performance [1,41]. PLSR addresses this limitation by computing latent variables that maximize covariance between explanatory features and the dependent variable, resulting in more accurate regression fitting [1,41]. PLSR is particularly suitable for high-dimensional regression problems with more parameters than samples, projecting predictors into a latent space to optimize outcomes [21]. Despite their advantages, both PCR and PLSR still require improvements in sensitivity and discrimination, especially for analyzing chemically similar mixtures.

Sazonova et al. [1] focused on detecting and predicting NT concentrations using voltammetry and pattern recognition techniques. They used PCR and PLSR to simultaneously detect DA and SE in in vitro mixtures. Data were collected by varying concentrations of DA and SE, creating 72 unique combinations. Measurements were taken using DPV over a range of potentials, resulting in over 1000 data points per mixture. The data were then analyzed using PCA to extract key features and PLSR to predict NT concentrations. Their results showed accuracies of 42–62% for DA and 33–50% for SE, which improved to 81–91% for DA and 91–100% for SE when extending the accuracy ranges. However, these results required expanding the acceptable concentration range, which indicated the predictions were not precise and deviated from true concentrations. Additionally, the models overfitted, as training accuracies exceeded testing accuracies. To improve prediction accuracy and avoid overfitting, further research should focus on simplifying NT recognition and refining feature selection for better concentration prediction.

Salimian et al. [33] used UV–vis spectroscopy and PCR to simultaneously detect levodopa and carbidopa in mixtures, drugs, and breast milk. Here, a UV–vis spectrophotometric approach was combined with two multivariate calibration techniques, namely, net analyte signal and PCR, to measure levodopa and carbidopa simultaneously in lab-prepared mixtures, pharmaceutical tablets, and breast milk samples. Using the net analyte signal method, the average recovery rates were found to be 98.10% for levodopa and 99.60% for carbidopa, with relative standard deviation values kept below 5.5% and 4%, respectively. In comparison, the PCR method yielded slightly lower recoveries: 96.86% for levodopa and 92.43% for carbidopa. To determine the optimal number of components in the PCR model, K-Fold cross-validation was applied, resulting in seven components for levodopa and three for carbidopa, with corresponding mean square error of prediction (MSEP) values of 1.50 and 7.14. Overall, the results showed that the net analyte signal model provided better performance than the PCR model. The net analyte signal-based method also proved effective for analyzing levodopa and carbidopa in both commercial tablet formulations and breast milk samples.

**Figure 11:**
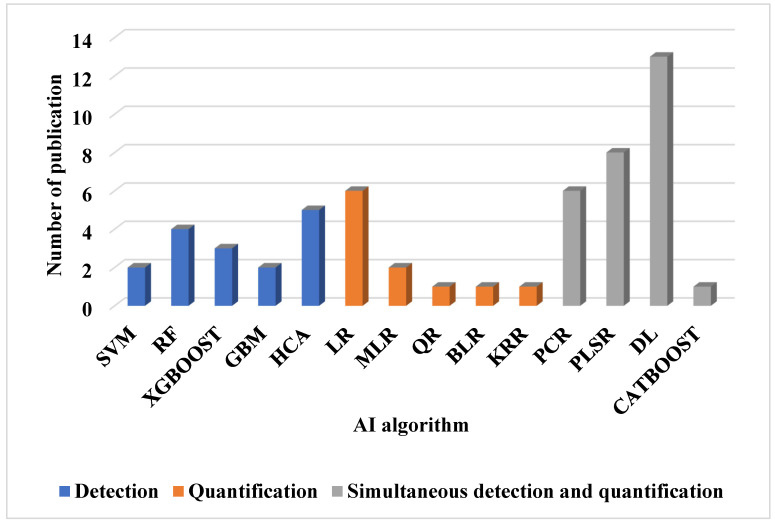
Frequency of publications using AI algorithms.

Nchouwat et al. [82] built upon the work done by Sazonova et al. [1] to detect NTs in complex mixtures and predict their concentrations with reduced procedural complexity for SE and DA. The average testing accuracies of estimation using PCA-GPR for DA alone, SE alone, and their mixture (DA–SE) were 87.6%, 88.1%, and 96.7%, respectively. Using PLS-GPR, the testing accuracies of estimation for DA alone, SE alone, and their mixture (DA–SE) were 87.3%, 83.8%, and 95.1%, respectively. Furthermore, they explored methods of reducing the procedural complexity in estimating NTs by finding reduced subsets of features to accurately detect and predict their concentrations. The reduced subsets of features found in the oxidation potential windows of the NTs improved the testing accuracy of the estimation of DA–SE to 97.4%. They showed that selecting scanning voltages found in the oxidation potential windows of these two NTs increased their estimation accuracies when true concentrations were used and did not need to increase the concentration levels for accuracy improvement, as achieved by Sazanova et al. [1]. Table 17 provides estimations of NTs, while Table 18 summarizes the algorithms used in the publications selected for this review, and Figure 11 shows a plot of the corresponding number of publications per AI algorithm used.

All publications with convolutional neural networks (CNNs), deep neural networks (DNNs), and artificial neural networks (ANNs) are placed in a single bin called deep learning (DL), as they are all similar techniques.

#### 4.3.2. Application of Deep Learning and Artificial Neural Networks

DL and ANNs are the most commonly used AI techniques for the biorecognition of NTs, with 13 publications (Figure 6). This is because deep learning avoids the complicated task of manual feature engineering, which is necessary for building AI models. Furthermore, DL techniques use 2D (image) data for AI model training and testing. These image data are readily acquired noninvasively using imaging techniques, both in vivo and in vitro, contrary to 1D data, which are mostly recorded invasively using voltammetric techniques and implantable biosensors. The biosensors need to be implanted into sites where biorecognition of the NTs is performed. Implantation of biosensors causes damage to the surrounding cells at the sites of implantation and biases the data recorded by the sensors. Furthermore, DL is more accurate than other AI techniques [42]. Additionally, the advantages of DL have favored the growth of interest in the simultaneous detection and quantification of NTs in recent decades. This is also due to the increase in computational power and memory of computers capable of handling 2D data.

The limitation on the use of DL is the need for extremely large 2D datasets, which are not always possible to obtain, especially from in vivo studies where animals are sometimes euthanized prior to data collection [76]. One-dimensional data are mostly analyzed using PCR and PLSR for the pattern recognition of NTs. This is because they are powerful pattern recognition algorithms made of efficient dimensionality reduction tools, such as PCA and partial least squares analysis (PLS), used in PCA and PLSR, respectively. These tools help to compute linearly independent features from 1D data. The features computed are then used for AI model training. This ensures that the trained models are not overfitted and can be generalized to other unseen 1D data [1].

Hoseok et al. [42] conducted the first study using DL to predict neurochemical concentrations from both in vitro and in vivo FSCV data, comparing its performance to PCR. They found that while DL and PCR performed similarly for detecting individual NTs (DA, EP, NE, and SE), DL outperformed PCR when detecting mixtures. Data were collected using FSCV across five concentrations (100–500 nM), with 500 scans per concentration, and data augmentation expanded the training set to 36,000 scans. DL achieved slightly higher accuracy (96.23%) than PCR (95.39%) for DA detection. The study demonstrated deep learning’s superiority in single-analyte detection but did not address simultaneous multi-analyte detection or practical in vivo application using DPV. Future work should focus on multi-NT detection using DPV for greater real-world applicability.

Seongtak et al. [43] developed a deep learning method to simultaneously estimate tonic levels of DA and SE with high temporal resolution in vitro. By leveraging data from FSCV, their approach addresses key limitations of traditional techniques, such as microdialysis, which struggle with low temporal resolution and are not well suited for monitoring multiple NTs over time. The deep learning model also demonstrated significantly greater accuracy than conventional background subtraction methods, especially in estimating SE concentrations. These findings highlight the potential of this approach for the real-time, simultaneous monitoring of both phasic and tonic NT activity in future in vivo studies.

#### 4.3.3. Application of Embedded Machine Learning

Sanjeet et al. [54] developed an optimized carbon thread-based miniaturized electrochemical sensing device capable of simultaneously detecting DA and SE, with the integration of machine learning to improve data analysis and reduce manual intervention. The device, fabricated using a CO_2_ laser scriber, demonstrated broad linear detection ranges (0.5–150 μM) for DA and 0.5–200 μM for SE, with low limits of detection (0.25 μM for DA, 0.22 μM for 5-HT) and strong linearity (R^2^ = 0.99 and 0.98, respectively; N = 3). Real sample testing in blood serum confirmed high recovery and selectivity. To enhance prediction accuracy, various ML regression models were applied to the analytical dataset (80% training; 20% testing). Among these, the decision tree, k-nearest neighbors, gradient boosting, adaptive boosting, and random forest models achieved R^2^ scores above 0.98 with low error rates, outperforming linear and support vector regression. Their study highlights the potential of combining miniaturized EC sensing with ML for highly accurate and efficient diagnostic applications.

This subsection analyzed how multiple NTs are simultaneously detected and classified into distinct categories and how their accurate quantities or concentrations in complex biological fluids can be predicted. The simultaneous detection and quantification of NTs is unique in that it entails the simultaneous estimation of multiple NTs in complex biological fluids, specifically targeting the discrimination of NT signals in the presence of interfering species like ascorbic acid, uric acid, metal ions, and other background noise. Furthermore, it has the potential to use data from various biosensors, including bioenzyme and nanoparticle-based implantable biosensors used for NT estimation. These sensors have enhanced sensitivity through the modification of their functional surfaces with chitosan-immobilized nanoparticles. Table 17 summarizes the reviewed works that focused on the estimation of NTs.

### 4.4. AI Algorithms and Voltammetric Techniques for NT Detection

As discussed in Section 3.2, voltammetric techniques in NT detection are advantageous over spectroscopy, fluorometry, and colorimetry because they can be easily implemented in vivo, are low cost, have rapid responses, and can easily be implemented in portable devices. The prominent works discussed and summarized in Table 19 include the state-of-the-art research published by Jose et al. [9] on the automatic detection of NTs and the state-of-the-art research published by Sazanova et al. [1], Hoseok et al. [42], and Nchouwat et al. [82] on the automatic detection and quantification of NTs.

### 4.5. Limitations of the Existing Studies Employing AI Algorithms Trained on Voltammetric Data

Several challenges persist in the accurate detection and quantification of neurotransmitters (NTs) in complex biological environments, as highlighted in current literature. These challenges include

1. Elevated detection thresholds for NT estimation: Sazonova et al. [1] extended the estimation thresholds of NT concentrations beyond the actual concentration ranges, resulting in wider confidence intervals. Consequently, the predicted NT concentrations often fail to reflect the true levels present in complex biological matrices, thereby compromising the reliability of these estimations.
2. Limited discrimination of NTs during simultaneous NT quantification: Although the proposed computational models demonstrate high performance metrics, they are frequently unable to fully discriminate between different NT species. Accurate and complete discrimination is essential for comprehensive profiling of individual NTs and is a critical requirement for identifying biomarkers associated with neurodegenerative diseases.
3. Restricted NT species coverage and simplified mixtures of NT species: Existing studies generally investigate only a limited subset of NTs, even though biological fluids naturally contain a vast array of NT species in dynamic equilibrium. For a more representative analysis, it is necessary to evaluate extended NT species libraries and more complex mixtures, which better reflect physiological conditions.
4. Resource-intensive computational models: Many of the AI-based approaches proposed for automatic NT detection and quantification are computationally intensive, resulting in slow processing times and high resource demands. These limitations hinder their applicability in real-time or near-real-time settings. To overcome this, model compression techniques such as knowledge distillation or transfer learning can be employed to develop lightweight, computationally efficient alternatives (e.g., simplified linear models).
5. Model overfitting and bias-related performance issues: Numerous AI models exhibit significant overfitting, as indicated by disproportionately high training accuracy compared to validation performance. This can be attributed to the high dimensionality, noise, and variance inherent in biological datasets. These issues cause models to learn noise patterns along with meaningful signals. Effective mitigation strategies include robust data preprocessing, noise reduction, and advanced feature engineering to isolate and prioritize relevant features from complex datasets.

### 4.6. Tools Used for the Automatic Detection and Quantification of Neurotransmitters

The need for the real-time monitoring of the “true” patterns of NTs in situ has caught the attention of researchers in the recent decade. This is due to the important roles played by the NTs in maintaining human physical and mental health. The monitoring of the NTs is very challenging due to the multiplexed signals and crosstalks generated by the NTs, especially in vivo. To this aim, numerous tools have been used to deconvolve the multiplexed signals and crosstalks recorded by electrochemical techniques. The tools reported in the 33 papers used for this review are both hardware and software tools. Two papers implemented deployable hardware devices. Concerning software tools, 2 papers used MATLAB versions R2009b and R2024b [1,36], one paper used Edge Impulse development platform [9] and the rest of the 30 papers used Python (versions 3.0 to 3.10) programming platforms including Jupyter Notebook (versions 4.0 to 6x), Google Collaboratory (accessed at various dates corresponding the when the experiments were done), R (versions 1.0.0 to 4.3.3) and VS Code (versions 1.0 to 1.90).

## 5. Discussion

The automatic detection and quantification of NTs can be classified as chemometric studies when analyses are performed in vitro or bioinformatic studies when analyses are performed in vivo. Chemometrics is the most reported, with 90% of the studies selected for this review performed in vitro. This is the application of AI in analytical chemistry and metabolomics. Chemometrics is a data-driven means of extracting valuable information from chemical systems for accurate decision-making. Chemometrics is inherently interdisciplinary, using methods frequently employed in core data-analytic disciplines, such as multivariate statistics, applied mathematics, and computer science, in order to address problems in chemistry. In summary, chemometrics serves as a vital toolbox for modern analytical chemistry, empowering scientists to extract actionable insights from complex chemical data. By harnessing the power of mathematical and statistical methods, chemometric experts contribute to advancements in various fields, including pharmaceuticals, environmental monitoring, food safety, and materials science. As analytical techniques continue to evolve and generate increasingly large datasets, the role of chemometrics in extracting knowledge from data is poised to become even more indispensable in the years to come.

### 5.1. Challenges Faced in the Automatic Detection and Quantification of Neurotransmitters

The automatic detection and quantification of NTs face many challenges that largely contribute to the degradation of the results obtained from chemometric or bioinformatic analyses. These challenges are included but not limited to the following:

1. The major challenge faced is the small size of the datasets available for the training of AI models. One reason for the small size of the datasets is the limitation in the range of operating potentials for some biosensors that are used to record the signal patterns of NTs. These limitations limit the maximum number of data points that can be recorded by a particular sensor. Another reason for the limited data is the need to euthanize animals prior to in vivo data recording. These problems can be solved by designing sensors with materials that remain stable over greater potential ranges and undertaking in vitro experiments to avoid the euthanizing of animals. Data augmentation techniques appropriate for these types of data should be explored to artificially increase the sizes of the datasets.
2. Implantable biosensors are very fragile and easily break during implantation or during manipulation while estimating NTs. This makes their use and maintenance very difficult. This difficulty may not be directly addressed by AI, but mechanically resistant nanofiber materials that are highly conductive can be used to mitigate this problem.
3. The surface modification of biosensors, aimed at enhancing their selectivity and sensitivity by using biopolymers to immobilize bioenzymes and nanoparticles, makes the sensors bulky. This bulkiness sometimes damages the tissues at their sites of implantation. The damaged tissues cause abscesses around the sensors, impeding electron diffusion between the sensors and the analytes under measurement. Furthermore, some biopolymers used as immobilizing matrices reject some neurochemicals. This is the case with chitosan rejecting ascorbic acid. These factors reduce the performance of the sensors and introduce noise to the measured signals. These challenges can be mitigated using AI by training models on larger, more representative datasets that include controlled noise to simulate the effects of surface modifications. This approach enhances the models’ ability to generalize to new, unseen data.
4. The passivation of biosensors greatly contributes to a reduction in their performance. This is due to the reduction in the sensitivity of the biosensors when a particular NT is adsorbed to the functional surface of the biosensors when used for a very long period. This problem can be mitigated through the use of bioenzymes and nanoparticles, which accelerate the conversion of NTs, thereby reducing their adsorption onto the functional surfaces of biosensors. Additionally, AI-based mitigation involves training models on larger and more representative datasets that incorporate controlled noise to simulate the effects of passivation. This strategy improves the models’ ability to generalize to new, unseen data.
5. Crosstalk and fouling of biosensors by NTs: These occur due to NTs’ similar electrochemical responses at certain potentials, as well as their comparable chemical structures to other neurochemicals and metal ions. These issues reduce the specificity and discriminative ability of the biosensors. This problem can be mitigated by designing biosensors that are highly specific to individual NTs using biocatalysts, including bioenzymes.
6. The effective surface areas of biosensors are not yet optimized for the detection of NTs in vivo. To this end, miniaturizing sensors by using nanofiber materials during sensor design will increase the effective contact surface area between the sensors and the analytes under study.

### 5.2. Potential Application of the Automatic Detection and Quantification of Neurotransmitters

NTs are present in various body fluids, including cerebrospinal fluids, blood, and urine. Simultaneous detection and quantification of NTs in these different fluids serve distinct purposes and present different challenges, as discussed below.

#### 5.2.1. Estimation of Neurotransmitters in Cerebrospinal Fluid from Brain Samples

**Figure 12:**
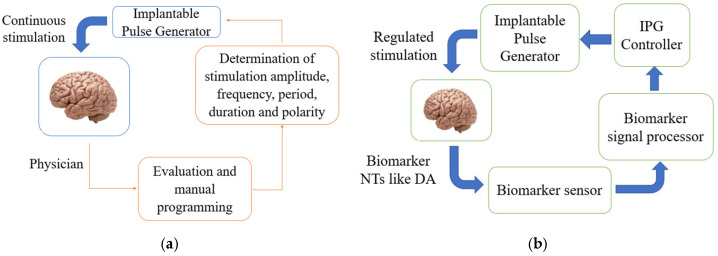
(**a**) Open-loop deep brain stimulation. A physician is needed to manually evaluate and determine the right signal parameters for the control of the IPG. (**b**) Closed-loop deep brain stimulation. No direct involvement of a physician in the evaluation and determination of the signal parameters for IPG control. The equilibrium concentration of DA at any time and location in the brain (body) determines its proper neurologic functions.

NTs present in the cerebrospinal fluid of the brain are important biomarkers for neurodegenerative diseases. For instance, DA is an important biomarker for Parkinson’s disease, while the interaction between SE and NEP in the brain plays an important role in anxiety and depression [92]. The U.S. Department of Health and Human Services declared the early detection and treatment of Parkinson’s disease to be national-priority tasks. This is because a 2022 Parkinson’s Foundation-backed study revealed that nearly 90,000 people are diagnosed with Parkinson’s disease every year in the U.S. and projected that over 1.2 million people in the U.S. will be living with Parkinson’s by 2030. According to the WHO, the global estimates in 2019 showed over 8.5 million individuals living with Parkinson’s disease, and 329,000 died from it [130]. Since dopamine is an important biomarker of Parkinson’s disease, the accurate real-time monitoring of the in vivo concentration of NTs and, hence, dopamine will help in the early diagnosis and treatment of Parkinson’s disease. Therefore, the potential application of the automatic detection and quantification of NTs and, hence, dopamine will be used in medical therapies such as deep brain stimulation for the treatment of neurological disorders, including Parkinson’s disease and depression. This will reduce the death tolls caused by neurological disorders [63,64,65,66,67]. The current therapeutic approach used in the treatment of neurological disorders is based on open-loop deep brain stimulation. The main drawback of the current open-loop deep brain stimulator is that it has no feedback loop to indicate the appropriate duration, intensity, and duty cycle of the electric impulses to be sent into the brain to stimulate the release of dopamine when there is a shortage of dopamine or when to stop sending electric pulses when dopamine is in excess [131]. As a result, electric impulses are sent nonstop and uncontrolled into the brain during the therapeutic treatment of Parkinson’s disease [132]. To remedy this, doctors usually depend on their experience to decide on the duration, intensity, and duty cycle of the electrical pulses to be administered to patients, which is not always accurate. Therefore, the models to be developed from the automatic detection and quantification of NTs will accurately and simultaneously enable the real-time measurement and monitoring of the signal patterns of several NTs in the brain [1]. Consequently, the early diagnosis of neurological diseases will be facilitated. Further, the models will enable the separation of the measured real-time signal patterns of the NTs to obtain discriminated signals for NTs, especially dopamine. The discriminated dopamine signals will then be fed back into the control loop of the open-loop deep brain stimulator to make it into a closed-loop deep brain stimulator. This new stimulator will enhance the treatment of Parkinson’s disease and other neurological diseases [131,132]. Human errors potentially made by doctors during the treatment of this disease will be reduced by indicating to the stimulator the appropriate electric signals to be sent into the brain during therapeutic treatments. Figure 12 summarizes the processes of open- and closed-loop deep brain stimulation therapies.

#### 5.2.2. Estimation of Neurotransmitters from Urine Samples

Accurately detecting and quantifying NTs in urine provides a simple way to monitor health and make specific medical diagnoses. Because it is easy to perform, urine testing is sometimes used in functional and integrative medicine to check for NT imbalance and nervous system function. In clinical settings, urinary metabolites, such as vanillylmandelic acid (VMA) for norepinephrine and homovanillic acid (HVA) for DA, help diagnose conditions like pheochromocytoma and neuroblastoma. They offer insights into how neurotransmitters are processed in the body. However, urinary NT levels do not directly relate to activity in the central nervous system (CNS). These levels reflect breakdown products from the body that are influenced by diet, kidney function, and liver metabolism. Therefore, their usefulness in standard psychiatric or neurological assessments is limited.

#### 5.2.3. Estimation of Neurotransmitters from Blood Samples

Measuring NTs in blood gives useful insights into how the nervous system works. It helps diagnose endocrine tumors, monitor drug therapy, and research biomarkers for neurological and psychiatric disorders. For instance, high blood serotonin levels may suggest carcinoid syndrome. Additionally, measuring DA or SE precursors in plasma can aid research on depression or Parkinson’s disease. In clinical trials, blood tests are used to track how well medications are working and whether patients are following their treatment plans. However, there are significant limitations to blood NT levels. The blood–brain barrier stops a direct link to central nervous system (CNS) activity. Also, circulating NTs usually exist in low amounts and break down quickly. These issues require sensitive testing methods that make blood testing more suitable for research and specific clinical uses rather than routine mental health assessments.

## 6. Conclusions

The significant problems faced by state-of-the-art biosensors that cannot be solved by conventional analytical methods are low sensitivity and selectivity among likely biological interfering NTs and background noise. Additionally, electrode “fouling” and crosstalk caused by the interference of signal patterns generated by NTs cannot be handled by conventional analytical methods. These problems are exacerbated when biosensors are used in complex biological fluids, including cerebrospinal fluids. Attempts to mitigate these problems have been made by modifying the functional surfaces of biosensors through the combined actions of nanoparticles and bioenzymes. However, lingering multiplexed signals and crosstalk resulting from interference between different species of NTs end up being recorded, especially in complex biological fluids. Therefore, AI algorithms have been proposed to efficiently deconvolve the lingering multiplexed signals and crosstalk by removing the background interference and noise to reveal the “true” signal patterns corresponding to NTs. This paper presented the first survey based on 33 peer-reviewed articles focusing on the application of AI algorithms for the automatic detection and quantification of NTs in complex matrices. The implementation of AI algorithms to data recorded by implantable biosensors has the potential to enhance the reliability of the biosensors in discriminating NT responses. This makes it possible for the “true” signal responses of NTs from multiplexed signals to be obtained. Real-time in situ discriminated responses, particularly those of DA, have potential applications in future therapies, including closed-loop deep brain stimulations, to control neurodegenerative diseases, including Parkinson’s disease, depression, and Alzheimer’s.

## Figures and Tables

**Table 1:** Summary of related reviews.

Paper	Summary	Scope			
		NT Analysis	Voltammetric Sensing Techniques	Simultaneous NT Detection	Application of AI
Bo Si et al. [4]	Summarized recent NT sensing techniques and explored prospects for their simultaneous detection			✓	
Yi Su et al. [6]	Analyzed current in vivo NT detection techniques, focusing on real-time brain disorder diagnosis	✓			
Saikat et al. [18]	Surveyed electrochemical techniques for real-time, high-temporal-resolution NT sensing.	✓	✓	✓	
Shimwe et al. [19]	Reviewed advances in NT detection, covering in vivo sampling, imaging, electrochemical, nano-sensing, spectrometric, and analytical methods for both in vitro and in vivo use	✓	✓	✓	
Yangguang et al. [23]	Highlighted key technical advances and in vivo NT studies that may aid brain disorder diagnostics	✓		✓	
Shiva et al. [25]	Examined how NTs interact with nanomaterials, their role in diagnostics, detection techniques, and prospects for simultaneous detection and use in biological samples	✓		✓	
Pathath et al. [77]	Summarized recent nanomaterial-based optical methods for dopamine detection, covering its clinical relevance and advances in spectroscopic techniques.		✓		
Xixian et al. [78]	Discussed dopamine detection via molecular recognition methods and recent advances using nanomaterials and molecularly imprinted polymers			✓	
Dunham et al. [79]	Reviewed electrochemical techniques for NT detection and studied depression mechanisms.		✓	✓	
This review	Reviews the use of AI for the automated detection and quantification of multiple NTs	✓	✓	✓	✓

**Table 2:** Inclusion and exclusion criteria adopted to select the articles for this review.

Criteria	Inclusion Criteria	Exclusion Criteria
Articles quality	Peer-reviewed research articles	Thesis and dissertations
Scope of the Articles	Articles with clear elaboration of the end-to-end AI algorithm used for the pattern recognition of NTs	Articles that do not meet this criterion
Publication Language	The articles must be published in English	Articles published in languages other than English
Research Index Terms	A subset of the following words must be addressed by the articles: pattern recognition, simultaneous detection and prediction, automatic quantification + neurotransmitters and machine learning, deep learning + chemometrics	Any article not falling into these subsets
Target neurochemicals	Articles analyzing neurotransmitters	Articles analyzing neurochemicals other than neurotransmitters
Detection limits of the NTs	Articles focusing on in vivo or in vitro analysis of NTs with concentrations within the physiological ranges	Ranges falling out the physiological ranges
Age of the publication	Articles published no earlier than the year 2009	Articles published earlier than the year 2009

**Table 4:** Summary of NTs studied in papers related to AI.

Category	Neurotransmitter	Chemical Formula	Localization	Role	Pathology	Reference
Indoleamine	Serotonin (SE)	C_10_H_12_N_2_O	Midbrain, hypothalamus, limbic system, cerebellum, pineal gland, spinal cord	Excitatory to other NTs, sleep, appetite, memory, cardiovascular regulation, temperature, walking	Nausea, headaches, regulation of mood, schizophrenia, anxiety, and depression	[1,3,40,41,42,43,44,45,46,52,53,54,55,56,57,58]
Catecholamines	Dopamine (DA)	C_8_H_11_NO_2_	Hypothalamus, substantia nigra	Excitatory to other NTs, pleasure, satisfaction, motivation, the regulation of emotional and social stress, learning, rewards, addictive behavior, motion control	Parkinson’s disease, Huntington’s disease, drug addiction, and schizophrenia	[1,2,3,9,24,35,36,37,38,40,41,42,43,44,46,47,48,49,50,51,52,53,54,55,57,58,59,60,61]
	Epinephrine (EP)	C_9_H_13_NO_3_	Tegmental and medulla	Fight-or-flight response	N/A	[3,34,35,37,42,46,52,58]
	Norepinephrine (NE)	C_8_H_11_NO_3_	Locus coeruleus of the midbrain, brain stem, limbic system, cerebral cortex, thalamus	Good feelings	Depression, ADHD, PTSD	[2,3,34,35,37,40,42,46,52,53,55,58]
Amino Acids	Glutamate (GL)	C_5_H_9_NO_4_	Central nervous system (brain and spinal cord)	Excitatory to other NTs, learning, memory, vision	Epilepsy, schizophrenia, excitotoxicity, multiple sclerosis, amyotrophic lateral sclerosis, Alzheimer’s disease, and Parkinson’s disease	[21,46,58,62]
	GABA	C_4_H_9_NO_2_	Hypothalamus, spinal cord, cerebellum, retina	Excitatory/inhibitory to other NTs	Epilepsy, convulsions, sleep disorders, pain, anxiety, autism, schizophrenia	[46,58]
Choline	Acetylcholine (ACH)	C_7_H_16_NO_2_^+^	Basal nuclei and cortex, neuromuscular junctions, brain	Excitatory to other NTs, memory, learning	Alzheimer’s disease, hallucinations, tetanic muscle spasms	[46,58]
Precursors	Levodopa	C_9_H_11_NO_4_	Oral drugs found in blood	Treatment of Parkinson’s disease	N/A	[2,3,33,41,45,46,58]

**Table 5:** Advantages of integrating AI with traditional biosensors for the estimation of NTs.

Aspect	Traditional Biosensors	AI-Integrated Biosensors
Functionality	Detect specific neurotransmitters based on chemical/biological reactions (e.g., enzyme-based, electrochemical sensors).	Use raw or preprocessed biosensor data as input for AI algorithms to enhance detection, classification, and interpretation of neurotransmitter signals.
Sensitivity and Accuracy	Good, but can suffer from noise, signal drift, and limited dynamic range.	Improved due to AI filtering, noise reduction, and pattern recognition. Better handling of low-concentration signals.
Real-Time Analysis	Limited; real time possible but often requires post-processing for complex analysis.	Real-time processing with AI models (e.g., neural networks) to instantly interpret complex patterns in neurotransmitter activity.
Data Processing	Mostly manual or using basic signal processing.	Automated, adaptive, and scalable using machine learning and deep learning.
Multi-Neurotransmitter Detection	Difficult; requires multiple sensors or complex setups.	Easier with AI models trained to distinguish overlapping signals from multiple neurotransmitters.
Adaptability	Fixed; often specific to a particular neurotransmitter and environment.	Adaptive; can learn and improve performance over time and adjust to new environments or analytes.
Complex Pattern Recognition	Poor; limited ability to recognize spatiotemporal patterns in neurotransmission.	Excellent; AI models can detect complex spatial and temporal trends in neurotransmitter dynamics.
Cost and Complexity	Generally lower cost, simpler design.	Higher development and computational costs due to AI integration and data infrastructure needs.
Hardware Requirements	Enzyme-based electrochemical sensors (e.g., for dopamine or serotonin).	AI-enhanced electrochemical arrays or optical sensors using deep learning to analyze neurotransmitter release patterns.

**Table 6:** Enhancement of biosensors for NTs estimation.

Target NT	Sensor Type	Performance-Enhancing Material	LOD and Dynamic Range	Summary	Reference
DA	∼100 μm amperometric enzyme-based carbon fiber microbiosensor	Tyrosinase- and ceria-based metal oxides immobilized by chitosan biopolymer	LOD of 1 nM, linear range of 10 nM to 220 μM, sensitivity of 14.2 nA·μM-1	Provides continuous, real-time monitoring of electrically stimulated dopamine release in the brain of an anesthetized rat.	[10]
L-Glutamate	60 µm amperometric enzyme sensor	Pt wire + GluOx + electropolymerized o-phenylenediamine barrier	LOD of ~0.3 µM	Lipid pre-coating + polymer barrier improved rejection of interfering species.	[104]
DA	EDTA-reduced graphene (EDTA-RG)	Graphene/Nafion composite on GCE	Interference from 1000× ascorbic acid was fully eliminated; high sensitivity	EDTA on graphene adds carboxyl groups; Nafion layer repels negatively charged interferents; improved ionic selectivity.	[105]
DA	Glassy carbon electrode	Perovskite LaFeO_3_ microspheres (nanospheres)	LOD of ~59 nM; linear range of 20 nM–1.6 µM	Electrocatalytic oxidation enhancement by the microspheres; effectively avoids interference from ascorbic acid/uric acid.	[106]
Dopamine and Serotonin	Enzymatic electrochemical platforms	Laccase/HRP immobilized on semiconducting polymer matrices	LOD of ~73 nM for DA and ~48 nM for SE	Use of novel semiconducting polymer matrices improved enzyme immobilization, stability, and selectivity (interferent effect ≤6.4%).	[107]
Dopamine	Graphene multitransistor array (gMTA)	Dopamine-specific DNA aptamer (EG–gFET)	LOD ~1 aM (10^−18^ M); dynamic range ~10 orders of magnitude up to 100 µM	Aptamer recognition + ultra-sensitive graphene FET transduction; tested in small-volume CSF and brain homogenate; high selectivity for DA.	[108]
Dopamine	Implantable aptamer-graphene microtransistors	Soft implantable aptamer–graphene microtransistor probe	Picomolar sensitivity in vivo; >19-fold selectivity for DA over norepinephrine	High-spatial-resolution (cellular-scale) real-time monitoring; aptamer specificity and microtransistor transduction deliver strong selectivity and sensitivity.	[109]
DA	Microelectrode	Gold disk and label-free electrochemical aptasensor	LOD of ~0.11 µM linear range of 0.5 µM–27 µM	Micro-electrode geometry (gold disk ~2 µm radius) + aptamer recognition improves spatial resolution and selectivity in brain slice environment.	[110]
DA, SE, EP	Wearable electrochemical aptasensor	CuMOF@InMOF heterostructure + AuNPs + thiolated aptamers	LODs: DA ~0.18 nM; SE ~0.33 nM; EP ~0.27 nM; dynamic range ~1 nM–10 µM	Multi-analyte simultaneous detection; MOF heterostructure + AuNPs enhance surface area and electron transfer; aptamers provide specificity; wearable format on sweat.	[111]
DA, SE, GL, ACH	Flexible multi-electrode array probe on polyimide substrate	PEDOT/GluOx and rGO/PEDOT/Nafion	LODs are GL: 0.0242 µM; ACH: 0.0351 µM; DA: 0.4743 µM and SE: 0.3568 µM	Multi-NT simultaneous detection; different electrode modifications tailored for each NT; selective coatings (e.g., Nafion to repel interferents) used for improved selectivity.	[112]

**Table 7:** Summary of electrochemical techniques for NT sensing in papers related to AI.

Detection Technique	Summary	Advantages	Limitations	References
CV	Performs redox processes by sweeping the potential of a working electrode linearly back and forth while measuring the resulting current.	➢ Provides detailed electrochemical information ➢ Simple setup ➢ Fast measurement	➢ Limited sensitivity for low NT levels ➢ Difficult in complex biological matrices due to overlapping signals	[40,44,50,52,54,55,56,57]
FSCV	Operates by applying a rapidly varying voltage to a microelectrode, enabling real-time monitoring of NT dynamics.	➢ Excellent temporal resolution (sub-second) ➢ Real-time NT monitoring ➢ High spatial resolution	➢ Requires specialized electrodes ➢ Potential fouling of electrode surface ➢ Limited to detecting electroactive NTs	[41,42,43,45,53,59,60,61]
DPV	Enhances electrode sensitivity by superimposing small voltage pulses onto a linearly increasing potential and measuring the current just before and after each pulse.	➢ High sensitivity and resolution ➢ Better at distinguishing close redox peaks ➢ Suitable for low concentration detection	➢ Requires optimized parameters for each analyte ➢ Can be influenced by interfering species in biological samples	[1,36,50,52,54]
SWV	Applies a symmetrical square wave potential on top of a staircase potential and measures the difference in current at the end of each forward and reverse pulse.	➢ Very high sensitivity ➢ Rapid data acquisition ➢ Good signal-to-noise ratio	➢ Complex data interpretation ➢ Susceptible to background noise if not properly controlled	[9,52]
AM	Applies a constant potential to an electrode and measures the resulting current proportional to the concentration of an electroactive species over time.	➢ Simple and direct measurement ➢ High temporal resolution	➢ Less selective; may detect multiple species ➢ Requires stable baseline	[48]
FL	Measures the intensity of fluorescent light emitted by a substance after it has absorbed light or other electromagnetic radiation.	➢ High sensitivity ➢ Suitable for imaging	➢ Requires fluorescent labeling ➢ Photobleaching and background fluorescence	[2,35,56]
CL	Measures the concentration of a substance by detecting the intensity of its color, typically using a colorimeter to quantify light absorption at a specific wavelength.	➢ Inexpensive ➢ Easy to use	➢ Low sensitivity ➢ Interference from colored species in sample	[3,37]
PH	Measures the intensity of light, typically in the visible spectrum, and is used to determine the concentration of substances based on the amount of light absorbed or transmitted through a sample.	➢ Simple and cost-effective ➢ Quantitative	➢ Low specificity ➢ Requires chromophore-labeled neurotransmitters	[51]
SP	Measures how much light a substance absorbs across a specific range of wavelengths, typically using a spectrophotometer.	➢ Quick and non-destructive ➢ Good for certain neurotransmitters	➢ Limited selectivity ➢ Overlap with other absorbing species	[33,46]
RS	Uses the scattering of monochromatic light, usually from a laser, to study vibrational, rotational, and other low-frequency modes in molecules.	➢ Label-free detection ➢ Molecular fingerprinting	➢ Weak signal ➢ Fluorescence interference ➢ Needs enhancement	[34]
MRS	Measures the magnetic properties of atomic nuclei to provide information about the chemical environment and structure of molecules in a sample.	➢ Noninvasive ➢ Real-time metabolic information ➢ Used in **clinical imaging** (e.g., GABA, glutamate)	➢ Low sensitivity ➢ High cost and specialized equipment	[21]
IM	Captures visual representations of NT signaling using specialized techniques like microscopy, MRI, CT scans, and fluorescence.	➢ Label-free ➢ Sensitive to binding events ➢ Works in complex samples	➢ Interpretation can be complex ➢ Slower than voltammetric methods	[38,47,49,58]

**Table 8:** Comparison of voltammetric techniques and other electrochemical techniques.

Aspect	Voltammetric Techniques	Other Electrochemical Techniques
Detection Method	Electrochemical voltammetry (e.g., CV, DPV, SWV)	Optical, piezoelectric, FET-based, or other non-electrochemical methods
Signal Type	Current vs. voltage curves (electrochemical signals)	Optical signals (fluorescence, absorbance), frequency shifts, etc.
AI Role	Pattern recognition, denoising, classification of voltammograms	Signal filtering, feature extraction, classification of non-electrical signals
Data Complexity	High-dimensional time series data, often noisy	Varies; can be image-based (optical), spectral, or frequency-based
Sensitivity to Neurotransmitters	High (μM to nM range), analyte-specific response curves	Also high, but depends on sensor type (e.g., optical = high specificity)
Real-Time Monitoring	Possible; commonly used for in vivo applications (e.g., FSCV)	Also possible; more common for wearable/portable biosensors
Miniaturization	Compatible with microelectrodes; widely used in implantable sensors	Compatible with lab-on-chip, wearable formats
Interference Handling	AI helps distinguish overlapping redox peaks of multiple neurotransmitters	AI separates mixed signals in optical or frequency domains

**Table 9:** Summary of the different AI learning algorithms in papers related to AI.

Learning Type	Definition	Type of Dada	Goal	Example of Tasks	Learning Signal	Common Algorithm	Human Effort for Labeling	Challenges	References
Supervised	Learning from fully labeled data	Labeled data	Predict outcomes (classification, regression)	Image classification, spam detection, price prediction	Direct supervision (ground-truth labels)	Linear regression, decision trees, SVM, neural nets	High	Requires large, labeled datasets	All 30 except [3,35,37]
Semi-supervised	Learning from a small amount of labeled and unlabeled data	Mostly unlabeled data with some labeled examples	Improve learning using a few labeled data points	Text classification with limited labels	Weak supervision (partial labels)	Self-training, co-training, label propagation	Moderate	Making good use of limited labeled data	N/A
Unsupervised	Learning from completely unlabeled data	Unlabeled data	Discover hidden patterns or structure	Clustering, dimensionality reduction	No supervision (structure learning)	K-means, PCA, DBSCAN, autoencoders	None	Interpreting unsupervised results	[3,35,37]
Reinforcement	Learning through interaction with an environment	States, actions, rewards	Maximize cumulative reward over time	Game playing, robotics, recommendation systems	Reward signal (positive/negative feedback)	Q-learning, SARSA, Deep Q-Networks (DQN), policy gradients	None (but requires environmental simulation)	Balancing exploration vs. exploitation	N/A

**Table 10:** Summary of the feature selection techniques.

Method	Type	Description	Pros	Cons	Examples
Statistical Tests	Filter	Selects features based on statistical scores	Fast and model independent	Ignores the interactions between features and is univariate	Chi-square, ANOVA, mutual information
Recursive Feature Elimination (RFE)	Wrapper	Recursively removes the least important features based on model performance	Considers feature interactions and is model-specific	Computationally expensive and prone to overfitting	RFE with SVM or RF
Forward/Backward Selection	Wrapper	Iteratively adds/removes features based on model score	Considers feature combinations	Slow with many features, model-specific	Stepwise regression
Lasso Regression	Embedded	Uses regularization to shrink less important features’ coefficients to zero	Integrated with model and handles multicollinearity	Only linear relationships with biased coefficients	L1 regularization
Tree-Based Feature Importance	Embedded	Uses importance-scores from models like decision trees	Captures nonlinear relationships and is fast with trees	Model-specific and sometimes less interpretable	Random forest, XGBoost
Filter + Wrapper or Embedded	Hybrid	Combines speed of filter with accuracy of wrapper/embedded methods	Balanced tradeoff between performance and efficiency	More complex implementation and tuning of parameters is required	SelectKBest + RFE

**Table 11:** Summary of data dimensionality reduction techniques.

Method	Type	Description	Pros	Cons	Use Cases	References
Principal Component Analysis (PCA)	Linear	Projects data into directions of maximum variance	Unsupervised, fast, and reduces redundancy	Assumes linearity, hard to interpret components	Preprocessing image compression	[1,21,24,33,37,42,46,48,57]
Linear Discriminant Analysis (LDA)	Linear	Finds axes that maximize class separation	Supervised, good for classification	Only works with labeled data, assumes normality	Face recognition, pattern classification	[2,3,35,37,49]
t-Distributed Stochastic Neighbor Embedding (t-SNE)	Nonlinear	Preserves local structure for visualization	Captures complex patterns and great for 2D/3D plots	Slow, non-deterministic, not suitable for downstream ML models	High-dimensional data visualization	[35]
Uniform Manifold Approximation and Projection (UMAP)	Nonlinear	Similar to t-SNE but faster and preserves more global structures	Fast, scalable, preserves global and local structures	Complex tuning, not always interpretable	Bioinformatics, NLP embeddings	[56]
Isomap	Manifold learning	Preserves geodesic distances on a manifold	Captures nonlinear structures	Sensitive to noise, slow on large datasets	Three-dimensional shape analysis and visualization	N/A
Neural Autoencoder	Autoencoder	Learns low-dimensional representations via neural networks	Learns nonlinear features and is customizable	Requires more data and tuning; it is a black-box, so less interpretable	Image denoising, anomaly detection	[9,35,43]
Truncated SVD	Matrix Factorization	Factorizes a matrix into low-rank approximations	Efficient, interpretable, works with sparse data	Assumes linearity, less powerful for nonlinear data	Text data recommender systems	[43]

**Table 12:** Feature categories extractable from NT voltammograms.

Feature Category	Features Extracted	Significance
Peak Features	Peak current, peak potential, peak separation, number of peaks, trough current, trough potential, number of troughs, trough separation	Fundamental for identifying redox processes and reaction reversibility
Statistical Features	Mean, median, standard deviation, variance, min, max, skewness, kurtosis	Quick signal summaries, useful for ML algorithms, and handles variability/noise
Shape Features	Slope before/after peaks, Peak symmetry, zero crossings around peak, Peak curvature, trough curvature	Capture redox shape details and good for distinguishing similar profiles
Area-Based Features	Area under the curve, peak area, integrated charge	Reflects total redox activity and good for quantifying analyte concentration
Signal Processing (Frequency Domain) Features	FFT coefficients, wavelet coefficients, entropy (spectral/sample), autocorrelation	Useful for advanced classification and helps handle noise or overlapping peaks

**Table 13:** Summary of AI model selection and hyperparameter tuning techniques.

Technique	Description	Use Case	Pros	Cons	References
Hold-Out Validation	Splits dataset into training and test sets, usually in a ratio of 80:20.	Quick checks large datasets	Simple and fast.	Performance depends on split and high variance.	[24,52,55,58]
K-Fold Cross-Validation	Splits data into K parts, trains on k-1, tests on 1, and repeats k times.	General-purpose model evaluation	Reduces variance, uses data efficiently.	Computationally expensive.	[1,2,9,24,33,34,35,40,46,47,49,53]
Stratified K-Fold Cross-Validation	Same as K-Fold but preserves class ratios.	Classification with imbalanced classes	Fairer evaluation in class imbalance.	More complex than regular K-Fold.	[24,111]
Leave-One-Out Cross-Validation	Special case of K-Fold where k = n (n is the number of samples).	Adapted for small datasets	Very low bias.	Very high variance and high computation cost.	[38,52]
Nested Cross-Validation	Inner loop for model tuning, outer loop for evaluation.	Hyperparameter tuning with fair evaluation.	Avoid overfitting during tuning.	Very computationally expensive.	[56]
Bootstrap	Samples data with replacement, evaluates across many resamples.	Adapted for small datasets, estimating confidence intervals.	Good variance estimation.	Biased estimates, complex interpretation.	N/A
Grid Search Cross-Validation	Exhaustively tries combinations of hyperparameters with cross-validation.	Hyperparameter tuning.	Systematic and thorough.	Computationally intensive and does not scale well.	[24,59,60]
Random Search Cross-Validation	Randomly samples hyperparameter combinations.	Tuning with large search spaces.	More efficient than grid search.	May miss optimal values.	[38]
Bayesian Optimization	Probabilistically selects promising hyperparameter values.	Advanced hyperparameter tuning.	More efficient and informed than grid/random search.	More complex implementation.	[24,82]
Automated ML (AutoML)	Uses meta-learning or optimization to automate model selection.	Users with limited ML expertise.	Handles selection, tuning, and ensemble techniques.	Less control; can be a black box.	[9,35,43]

**Table 16:** Motivation for AI integration with traditional electrochemical biosensors.

Challenge	Effect on Feature Extraction	Impact on Training Robustness and Model Generalization	Motivation for AI Integration
Peak Overlap of NT voltammograms	Overlapping oxidation/reduction peaks from multiple neurotransmitters make it difficult to isolate distinct analytical features (e.g., peak height, area, or position) using conventional algorithms.	Models trained on idealized or separated peaks fail to generalize to real signals where peaks merge or distort, reducing classification accuracy.	AI models (e.g., machine learning, deep learning, CNNs) can learn nonlinear representations that deconvolve overlapping signals and extract compound-specific patterns without explicit peak separation.
Background Current Drift	Time-dependent baseline fluctuations due to electrode fouling, temperature changes, or electrolyte instability obscure true faradaic currents, complicating baseline correction and noise filtering	Drift introduces inconsistent feature scaling across sessions, leading to poor model robustness and domain transfer between experiments or electrodes.	Adaptive AI methods can learn to separate drift components from meaningful electrochemical signals and maintain stable representations across time and devices.
Redox Potential Shift	Variations in redox peak position due to electrode surface chemistry or environmental factors distort fixed-parameter feature extraction routines (e.g., fixed voltage windows).	Models trained on data from one electrode or environment perform poorly when applied to others with shifted potentials, limiting generalization.	AI models can learn invariant representations that adapt to potential shifts, aligning features across conditions and enabling cross-device or cross-subject transferability.

**Table 17:** Summary of the estimations of reviewed papers related to AI.

Scope	Summary	Advantages	Disadvantages	References
NT detection	These are classification processes that group NTs one at a time into distinct categories without prior knowledge of their types or concentrations in the biological fluid being studied.	➢ Confirms presence or absence of neurotransmitters ➢ Useful for qualitative analysis ➢ Simpler and less data-intensive	➢ Lack of quantitative information ➢ Cannot assess changes in neurotransmitter levels ➢ Limits clinical or pharmacological relevance	[2,3,9,35,37,38,40,44,47,48,49,50,52,56,59,60,61]
NT quantification	These are regression processes used to quantify NT concentrations one at a time, based on prior knowledge of the types of NTs present in the biological fluid being studied.	➢ Allows for modeling and estimation based on indirect data (e.g., behavior, imaging) ➢ Can be used when direct measurement isn’t possible	➢ Risk of inaccurate results if detection fails ➢ Predictions may lack biological validation ➢ Depends heavily on quality of input data	[21,24,34,36,45,46,47,50,52,53,56]
Simultaneous Detection and quantification of NTs	These are combined classification and regression processes in which multiple NTs are simultaneously categorized and their concentrations quantified, without prior knowledge of the types or concentrations of NTs present in the biological fluid being studied.	➢ Combines strengths of both detection and prediction ➢ Enables real-time, data-driven decision-making ➢ High clinical and research utility	➢ Technically complex and computationally intensive ➢ Requires advanced instrumentation and algorithms ➢ May be expensive or resource-demanding	[1,33,42,43,54,55,57,58,82]

**Table 18:** Summary of the AI algorithms used for NT estimation.

Algorithm	Learning Type	Summary	References
LDA	Supervised	Reduces data dimensions while maximizing class separation by finding the feature combinations that best distinguish between categories.	[2,3,35,37,49]
SVM	Supervised	Finds the optimal boundary (hyperplane) to separate classes by maximizing the margin between different class data points for better generalization.	[38,55]
RF	Supervised and ensemble	Builds multiple decision trees and combines their outputs for more accurate, performant, robust, and stable predictions.	[38,52,55,56]
GBM and CATBOOST	Supervised and ensemble	Builds models sequentially, where each new model corrects errors made by the previous ones and combines many weak learners (usually decision trees) to create a strong predictive model.	[24,41,48]
XGBOOST	Supervised	An optimized version of gradient boosting that is faster and more efficient through advanced regularization, parallel processing, and handling of missing values.	[40,48,49]
HCA	Unsupervised	Builds a hierarchy of clusters by either merging or splitting data points based on similarity and creates a dendrogram to visualize the nested grouping of data.	[2,3,35,37,55]
LR	Supervised	Models the relationship between one independent variable and one dependent variable by fitting a straight line and predicts the dependent variable based on the linear relationship with the independent variable.	[46,50,51,52,55,56]
MLR	Supervised	Models the relationship between two or more independent variables and one dependent variable, fitting a linear equation to predict the outcome based on the combined effect of all input variables.	[35,37]
QR	Supervised	Models the relationship between the independent variable(s) and the dependent variable using a second-degree polynomial (a quadratic equation).	[46]
BLR	Supervised	Incorporates Bayesian inference to estimate the distribution of model parameters, providing a probabilistic approach to linear regression and offering not just point estimates but also uncertainty estimates for the model’s predictions.	[36]
KRR	Supervised	Combines ridge regression with the kernel trick to model nonlinear relationships and maps input features into a higher-dimensional space to perform linear regression in that space, enabling it to capture complex patterns.	[36]
PCR	Supervised and dimensionality reduction	Combines principal component analysis (PCA) for dimensionality reduction with linear regression. It uses the principal components (uncorrelated features) as inputs to predict the target variable.	[1,33,41,42,82]
PLSR	Supervised and dimensionality reduction	Reduces predictors to a smaller set of uncorrelated components while maximizing the covariance between predictors and the response variable.	[1,21,34,41,46,57,126]
DL	Supervised, semi-supervised, unsupervised, or reinforcement learning	Uses multilayered neural networks to automatically learn complex patterns from large amounts of data.	[9,35,37,42,43,45,47,53,58,59,60,61]

**Table 19:** Studies integrating AI with voltammetry for the detection of NTs.

Study	ML Algorithm	NTs	Conc. Range	Sensing Technique	Dataset Measurements	Max. Acc.%	Type of Study
Sazanova et al. [1]	PCR, PLSR	DA, SE	0–100 (uM)	DPV	216 Cross-validation	100 (with extended true values)	Simultaneous detection and quantification
Jose et al. [9]	TinyML (DL)	AA, UA, DA, AA/DA, UA/DA, AA/UA/DA	0–500 (uM)	SWV	5492 (augmented) 80:20 split	98.1	Detection
Hoseok et al. [42]	DL, PCR	DA, SE, EP, NE	0–700 (nM)	FSCV	36,000 (augmented) 50:50 split	96.23	Simultaneous detection and quantification
Nchouwat et al. [82]	PCR, PLSR	DA, SE	0–100 (uM)	DPV	216 Cross-validation	98 (with true values)	Simultaneous detection and quantification

## Data Availability

No new data was created or analyzed in this study.
